# Identification of hypoxia-related diagnostic biomarkers and immune signatures in diminished ovarian reserve

**DOI:** 10.3389/fgene.2025.1626992

**Published:** 2025-08-04

**Authors:** Minxue Song, Lili Ni, Zebing Ma, Xin Zhong, Yibing Liu, Jilin Kuang, Ping Li

**Affiliations:** ^1^ Department of Gynecology, The Second Affiliated Hospital of Hunan University of Chinese Medicine, Changsha, Hunan, China; ^2^ Department of Orthopedics, The Second Affiliated Hospital of Hunan University of Chinese Medicine, Changsha, Hunan, China; ^3^ Graduate School, Hunan University of Chinese Medicine, Changsha, Hunan, China

**Keywords:** diminished ovarian reserve, hypoxia-related gene, high ovarian reserve, low ovarian reserve, immune cell infiltration

## Abstract

**Background:**

Diminished ovarian reserve (DOR) becomes more common with age, and hypoxia is a key cause of apoptosis in ovarian granulosa cells. This study investigated the genetic links between hypoxia and DOR.

**Methods:**

The GSE87201 dataset for DOR was sourced from Gene Expression Omnibus database, normalized for common differentially expressed genes (Co-DEGs), and identified Hypoxia-related differentially expressed genes (HRDEGs) *via* GeneCards; Receiver Operating Characteristic (ROC) curves evaluated HRDEGs’ diagnostic value, and protein-protein interaction networks were visualized with STRING and Cytoscape. Enrichment analyses included Gene Ontology (GO) and Kyoto Encyclopedia of Genes and Genomes (KEGG) pathways, and immune cell infiltration compared ovarian reserve groups. A granulosa cell injury model was created using 4-hydroperoxycyclophosphamide (4-HC), with Quantitative real-time PCR and Western blot measuring FANCI and KAT2A expression, and Cell Counting Kit-8 assays and flow cytometry assessing cell proliferation and apoptosis.

**Results:**

Twelve hypoxia-related genes were differentially expressed between low ovarian reserve (LOR) and high ovarian reserve (HOR), with 17 linked to DOR; eight pathways differed between LOR and HOR. Six hub genes (*FANCI*, *KAT2A*, *TACC3*, *TPX2*, *VHL*, *WSB1*) were enriched in Fanconi anemia and HIF-1 pathways, affecting microtubules, spindle formation, and cytoskeleton dynamics during mitosis. Immune cell infiltration analysis showed significant differences, with *FANCI*, *TACC3*, and *TPX2* correlating with immune populations. The DOR group had increased *FANCI* and *KAT2A* levels compared to Control (two of the several genes that were matched were randomly selected for validation), alongside reduced cell viability and increased apoptosis.

**Conclusion:**

*FANCI*, *KAT2A*, *TACC3*, *TPX2*, *VHL*, and *WSB1* may be diagnostic biomarkers for DOR, providing novel insights for future research into the pathogenesis of hypoxia-induced DOR.

## 1 Introduction

Diminished ovarian reserve (DOR) is characterized by a reduction in both the quantity and quality of oocytes within the ovaries. This condition is frequently associated with decreased levels of anti-Müllerian hormone, lower antral follicle counts, and elevated follicle-stimulating hormone levels, ultimately resulting in reduced fertility among women (Pastore et al., 2018). Data from the Society for Assisted Reproductive Technology Clinical Outcomes Reporting System indicate an upward trend in the incidence of DOR, increasing from 19% to 26%, and increasingly affecting younger women (De La Garza et al., 2005). Notably, the incidence among women under the age of 40 has reached 42% (Devine et al., 2015). DOR commonly presents with physical symptoms such as irregular menstrual cycles and secondary amenorrhea and is often accompanied by psychological issues, including insomnia and anxiety (Giovanale et al., 2015). Furthermore, DOR poses a risk of progressing to premature ovarian failure, which can significantly negatively impact fertility and overall family wellbeing (Liang et al., 2021). Current medical interventions primarily aim to regulate the hypothalamic-pituitary-ovarian axis through the administration of artificial hormones to restore normal reproductive endocrine function. This approach, however, is constrained by the potential adverse effects of hormone therapy and its failure to address the underlying causes of the condition.

Hypoxia has been recognized as a significant factor contributing to apoptosis in ovarian granulosa cells, which can result in follicular atresia and a reduction in oocyte numbers, thereby exacerbating ovarian decline (Yadav et al., 2019). Granulosa cells create a microenvironment conducive to oocyte development and play a regulatory role in follicular development, akin to oocytes (Jiao et al., 2021). Under hypoxic conditions, there is an increase in intracellular reactive oxygen species, which intensifies oxidative stress and induces modifications in proteins, lipids, and DNA. These changes can lead to autophagy in granulosa cells and oocytes (Aiken et al., 2019). Recent studies have demonstrated that hypoxia-induced germ cell death can precipitate early amenorrhea in mammals, resulting in the depletion of follicular reserves in mice and the inhibition of ovarian luteinizing cell production in bovines, as well as impaired follicular cell development (Klipper et al., 2010; Winship et al., 2022). Although hypoxia-associated genes have been implicated in various diseases, including ovarian cancer and polycystic ovarian syndrome, their role in the diagnosis and treatment of DOR remains inadequately understood.

The objective of this study was to investigate the potential genetic mechanisms associated with hypoxia and DOR. Bioinformatics approaches have been extensively utilized to elucidate the potential mechanisms of genes and diseases (Fu et al., 2020). We acquired the DOR GSE87201 (Barragan et al., 2017) dataset from the Gene Expression Omnibus (GEO) database and conducted normalization and intergroup differential analyses to identify common differentially expressed genes (Co-DEGs). Hypoxia-related differentially expressed genes (HRDEGs) were extracted from the GeneCards database following differential analysis using the DOR dataset. The diagnostic efficacy of HRDEGs for patients with DOR was evaluated by constructing receiver operating characteristic (ROC) curves. Protein–protein interaction (PPI) networks and visualization analyses were developed using the Search Tool for the Retrieval of Interacting Genes (STRING) database and the Cytoscape software. Subsequently, we performed Gene Ontology (GO) and Kyoto Encyclopedia of Genes and Genomes (KEGG) pathway enrichment analyses to elucidate the biological functions and pathways involved. Furthermore, immune cell infiltration analysis was conducted to verify differential expression between high ovarian reserve (HOR) and low ovarian reserve (LOR) groups. The expression and function of key genes associated with DOR were further validated using a granulosa cell injury model induced by 4-hydroxycyclophosphamide (4-HC). The *in vitro* experiments demonstrated that silencing of *FANCI* and *KAT2A* significantly reduced apoptosis and restored cell viability and cell cycle progression in injured granulosa cells, thereby confirming their pathogenic role in hypoxia-induced ovarian dysfunction. Our thorough and critical analysis uncovered several novel hub genes, specifically *FANCI* and *KAT2A*, along with distinct pathways such as Fanconi anemia and Notch signaling, which had not been previously implicated in earlier studies conducted on this particular dataset, highlighting the potential for new insights and understandings in this area of research.

## 2 Materials and methods

### 2.1 Collecting and preprocessing data

**FIGURE 1:**
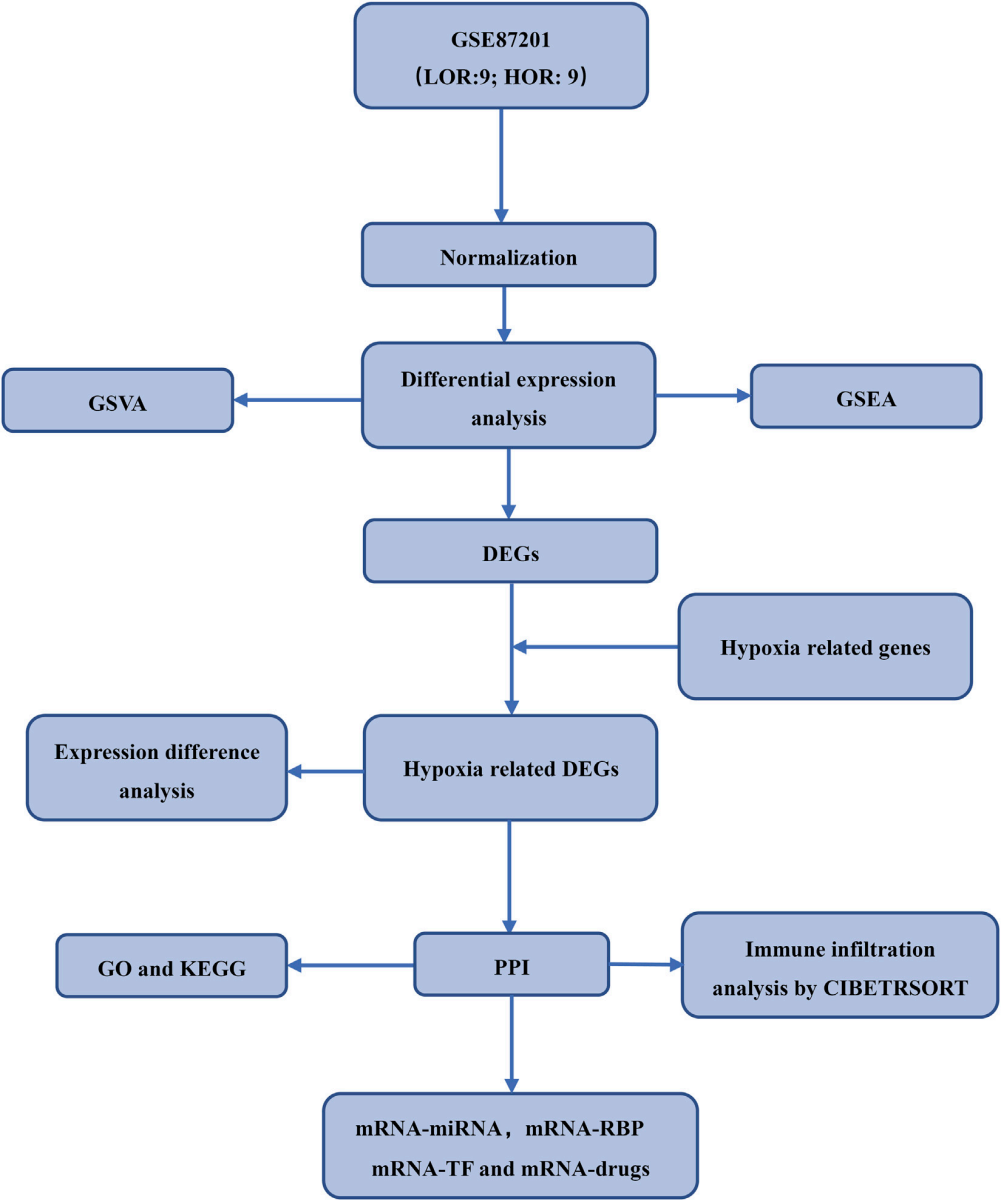
Study flow chart.

The expression profiling dataset GSE87201 for *Homo sapiens*, pertinent to DOR, was retrieved from the GEO database (Barrett et al., 2007) utilizing the R package GEOquery (Davis and Meltzer, 2007). The dataset comprised thirty-five samples, categorized into human oocytes from individuals aged 20–22 years and 33–35 years. Specifically, the 20–22 age group included seven samples with low ovarian reserve (LOR) and 9 with high ovarian reserve (HOR), while the 33–35 age group comprised nine LOR and nine HOR samples. The dataset probes were annotated using the microarray platform files GPL17586 to incorporate expression profiling data from the LOR and HOR samples aged 33–35 years for subsequent analyses. Hypoxia-related genes were identified using “hypoxia” as a search term in the GeneCards database (https://www.GeneCards.org/) and corroborated by articles published in PubMed. Supplementary Tables S1 and S2 provide a comprehensive list of genes implicated in DOR and hypoxia. This study utilized exclusively publicly available de-identified genomic data from the GEO database (accession GSE87201). No primary human or animal samples were collected by the authors. The original data generation by Barragán et al. obtained appropriate ethical oversight as stated in their publication (Barragan et al., 2017), including Institutional Review Board approval and participant informed consent. To account for potential batch effects across sample processing batches, the ComBat function from the R package was applied to the normalized expression matrix. Batch correction was performed prior to downstream differential expression and PCA analysis. The effectiveness of batch effect removal was evaluated using principal component analysis (PCA), comparing sample clustering before and after correction. The experimental workflow is depicted in Figure 1.

### 2.2 Normalization of DOR-related differential expression genes

The GSE87201 dataset was standardized utilizing the R package limma (Ritchie et al., 2015), applying a threshold of |log fold change (FC)| > 1 and *P* < 0.05. Subsequently, the dataset underwent standardization and differential expression analysis to identify differentially expressed genes (DEGs) between LOR and HOR conditions. Volcano plots were generated to visualize upregulated genes (logFC >0) and downregulated genes (logFC <0). The standardized dataset was further analyzed for differential expression, identifying Co-DEGs between LOR and HOR, which were then intersected with hypoxia-related genes to derive HRDEGs. Venn diagrams were employed to illustrate the HRDEGs. The outcomes of the differential expression analysis were visualized through volcano plots and heatmaps, created using the ggplot2 and pheatmap packages in R.

### 2.3 Gene set enrichment analysis (GSEA) and gene set variation analysis (GSVA)

Genes within the GSE87201 dataset were categorized into two groups based on the sign of their logFC values. GSEA was conducted on the DEGs using the R package clusterProfiler, with significance criteria set at p. adj <0.05 and a false discovery rate (FDR) *q*-value <0.25 (Subramanian et al., 2005; Wu T. et al., 2022; Liu et al., 2023). All genes within the LOR and HOR regions were subjected to analysis utilizing GSVA enrichment (Kowalski et al., 2016). The screening criteria employed were a p-value of less than 0.05 and an absolute log fold change (|logFC|) greater than 0.5, aimed at investigating biological processes, cellular composition, and molecular functions (MF). GSEA and GSVA were conducted on the entire gene expression dataset rather than DEGs alone, to capture pathway-level changes that may arise from coordinated but modest shifts in gene expression, in line with established best practices.

### 2.4 Construction of the ROC

ROC curves for the HRDEGs were generated using the pROC package in R software through univariate logistic regression models (Mandrekar, 2010). For each HRDEGs, gene expression levels served as the predictor variable, with ovarian reserve status (LOR/HOR) as the binary outcome. Predicted probabilities derived from these models were used to construct ROC curves. The area under the curve (AUC) was computed to evaluate the diagnostic efficacy of individual HRDEGs for DOR, with 95% confidence intervals calculated *via* 2,000 bootstrap resamples. AUC values range from 0.5 to 1, where values approaching one indicate superior diagnostic performance: AUC 0.5–0.7 denotes low accuracy, 0.7–0.9 moderate accuracy, and >0.9 high accuracy.

### 2.5 Protein–protein interaction (PPI) and messenger ribonucleic acid regulation network

PPI analysis of HRDEGs was performed using the Search Tool for the Retrieval of Interacting Genes (STRING) database (Szklarczyk et al., 2019), with the confidence level set to medium (0.400). Cytoscape (Shannon et al., 2003) (version 3.9.1) was employed to visualize the PPI network model, designating HRDEGs as hub genes.

The miRDB database (Chen and Wang, 2020) was utilized to predict micro-ribonucleic acids (miRNAs) that interact with these hub genes. The interaction network between messenger ribonucleic acid (mRNA) and miRNA was constructed by extracting segments of the database with a target score exceeding 80. The prediction of RNA binding protein (RBP) interactions with hub genes was performed using the ENCORI database (https://starbase.sysu.edu.cn/) (Li et al., 2014). The mRNA–RBP interaction network was then delineated with parameters set at ClusterNum > 6 and clipExpNum > 6. Transcription factors (TFs) interacting with hub genes were identified through the CHIPBase database (version 3.0) (https://rna.sysu.edu.cn/chipbase/) (Zhou et al., 2017) and the hTFtarget database (http://bioinfo.life.hust.edu.cn/hTFtarget) (Zhang et al., 2020). Furthermore, potential drugs or small molecules interacting with the hub genes were identified *via* the Comparative Toxicogenomics Database (CTD) (http://ctdbase.org/) (Davis et al., 2021), and the mRNA-TF and mRNA–drug interaction networks were visualized using Cytoscape software.

### 2.6 GO and KEGG pathway enrichment analyses

The GO (Gaudet et al., 2021) and KEGG (Kanehisa et al., 2017) analyses are extensively utilized databases for the storage of genomic, biological pathway, disease, and drug information. GO annotation analysis of hub genes was conducted using the R package clusterProfiler (Yu et al., 2012). The criteria for entry screening were set at a p-adjusted value of less than 0.1 and an FDR q-value of less than 0.05.

### 2.7 Immune cell infiltration analysis of HRDEGs

The matrix data from the DOR datasets, specifically LOR and HOR, were analyzed for immune cell enrichment scores greater than zero utilizing the CIBERSORT package (Newman et al., 2015) in conjunction with the LM22 feature gene matrix. This approach facilitated the acquisition and presentation of specific results pertaining to the immune cell infiltration abundance matrix. Correlations between various immune cells within the LOR and HOR datasets were visualized using the Spearman algorithm and the R package ggplot2. Additionally, correlations between immune cells and key genes were determined by integrating the gene expression matrix from the DOR dataset, which enabled the creation of hotspot maps *via* the R package ggplot2.

### 2.8 Modeling of granulocyte injury

KGN cells were seeded in 96-well plates at a density of 5 × 10^5^ cells per well and incubated overnight to allow for wall attachment. Following passaging and cell attachment, 4-HC was introduced into the target culture medium at final concentrations of 0.2, 2.0, 6.0, and 10.0 μmol/L, and the cells were subsequently mixed and incubated for 48 h. Cell viability was assessed using the CCK-8 assay, while apoptosis was evaluated using Annexin-V-FITC/PI-PE staining. This process facilitated the identification of optimal concentrations of 4-HC for the model of 4-HC-induced granulosa cell response.

### 2.9 CCK8

In this study, distinct groups of cells in the logarithmic growth phase were seeded at a density of 2,000 cells per well in 96-well plates. The cells were incubated at 37°C in a humidified atmosphere containing 5% CO_2_ for 6 hours to allow for adhesion to the well surfaces. Following attachment, various treatments were administered according to the designated experimental groups, with six replicate wells established for each condition. Two hours prior to the conclusion of the incubation period, 10 μL of CCK-8 solution was added to each well. At the end of the incubation, optical density at 450 nm (OD450) was measured using a microplate reader.

### 2.10 Detection of apoptosis and cell cycle by flow cytometry

Subsequently, the cell suspension was transferred to a centrifuge tube containing an appropriate volume of culture medium, centrifuged at 1,000 rpm for 5 min, and the supernatant was discarded. The cell pellet was washed once with phosphate-buffered saline (PBS). Annexin V Binding Solution was then added to achieve a final cell concentration of 1 × 10^6^ cells/mL. The cell suspension was mixed with Annexin V-FITC conjugate and propidium iodide (PI) solution, followed by the addition of 1X Annexin V Binding Solution. The samples were analyzed using flow cytometry within 1 hour.

For further analysis, cells were cultured in 6-well plates until they reached 60%–70% confluence. The cells were washed twice with PBS, collected, fixed, and subsequently pelleted. Approximately 1 mL of ice-cold PBS was added to resuspend the cells. Centrifuge the cells once more and carefully remove the supernatant, ensuring approximately 50 μL of PBS remains to prevent cell aspiration. Gently tap the bottom of the centrifuge tube to adequately disperse the cells and prevent clumping. For optimal staining results, it is recommended to complete the flow cytometry assay within 24 h. Red fluorescence should be detected at an excitation wavelength of 488 nm using flow cytometry, concurrently measuring light scattering.

### 2.11 Quantitative real-time PCR (qRT-PCR) assay

Total RNA was extracted from tissue samples utilizing TRIzol reagent (Invitrogen) and quantified *via* NanoDrop spectrophotometry. High-quality RNA was subsequently reverse-transcribed into complementary DNA (cDNA) employing the iScript™ cDNA Synthesis Kit (Bio-Rad) in accordance with the manufacturer’s instructions. The qRT-PCR was conducted using SYBR^®^ Green Master Mix (Applied Biosystems) on a StepOnePlus™ Real-Time PCR System. The reaction mixture, with a total volume of 20 μL, comprised 1 µL of cDNA, 10 µL of SYBR Green Master Mix, and 0.5 µM of each primer. The cycling conditions were as follows: initial denaturation at 95°C for 10 min, followed by 40 cycles of 95°C for 15 s and 60°C for 1 min. Specificity was verified through melting curve analysis. Primer sequences were designed utilizing Primer-BLAST software and subsequently validated for efficiency. The expression levels of target genes were normalized to GAPDH employing the 2^−ΔΔCt^ method. Each sample was analyzed in triplicate.

### 2.12 Western blotting

Proteins were extracted from tissues or cells using RIPA buffer supplemented with protease inhibitors. Protein concentrations were quantified using the BCA Protein Assay Kit. Equal amounts of protein were resolved by SDS-PAGE on 10% polyacrylamide gels and transferred onto PVDF membranes. Membranes were blocked with 5% non-fat milk in TBST for 1 hour at room temperature, followed by overnight incubation at 4°C with primary antibodies against the target proteins at a 1:1,000 dilution. Following washing, membranes were incubated with HRP-conjugated secondary antibodies at a 1:5,000 dilution for 1 hour at room temperature. Detection of signals was performed using an ECL chemiluminescence kit, and imaging was conducted with a ChemiDoc imaging system. β-actin served as an internal control to normalize protein loading. Each experiment was conducted at least three times to ensure reproducibility.

### 2.13 Statistical methods

The data analysis was rigorously performed utilizing R software (4.3.3), a robust platform for statistical computing. Continuous variables were reported as means with their corresponding standard deviations, offering a comprehensive depiction of the data’s variability. The Wilcoxon rank-sum test was applied to compare different groups, thereby accommodating non-parametric data distributions. Additionally, all results not explicitly specified were calculated using the Spearman test, which is particularly advantageous for evaluating correlations among ranked variables. A p-value of less than 0.05 was considered statistically significant, suggesting that any observed differences or associations were unlikely to be due to random chance, thereby enhancing the credibility of the findings.

## 3 Results

### 3.1 Processing of the DOR dataset and identification of HRDEGs

**FIGURE 2:**
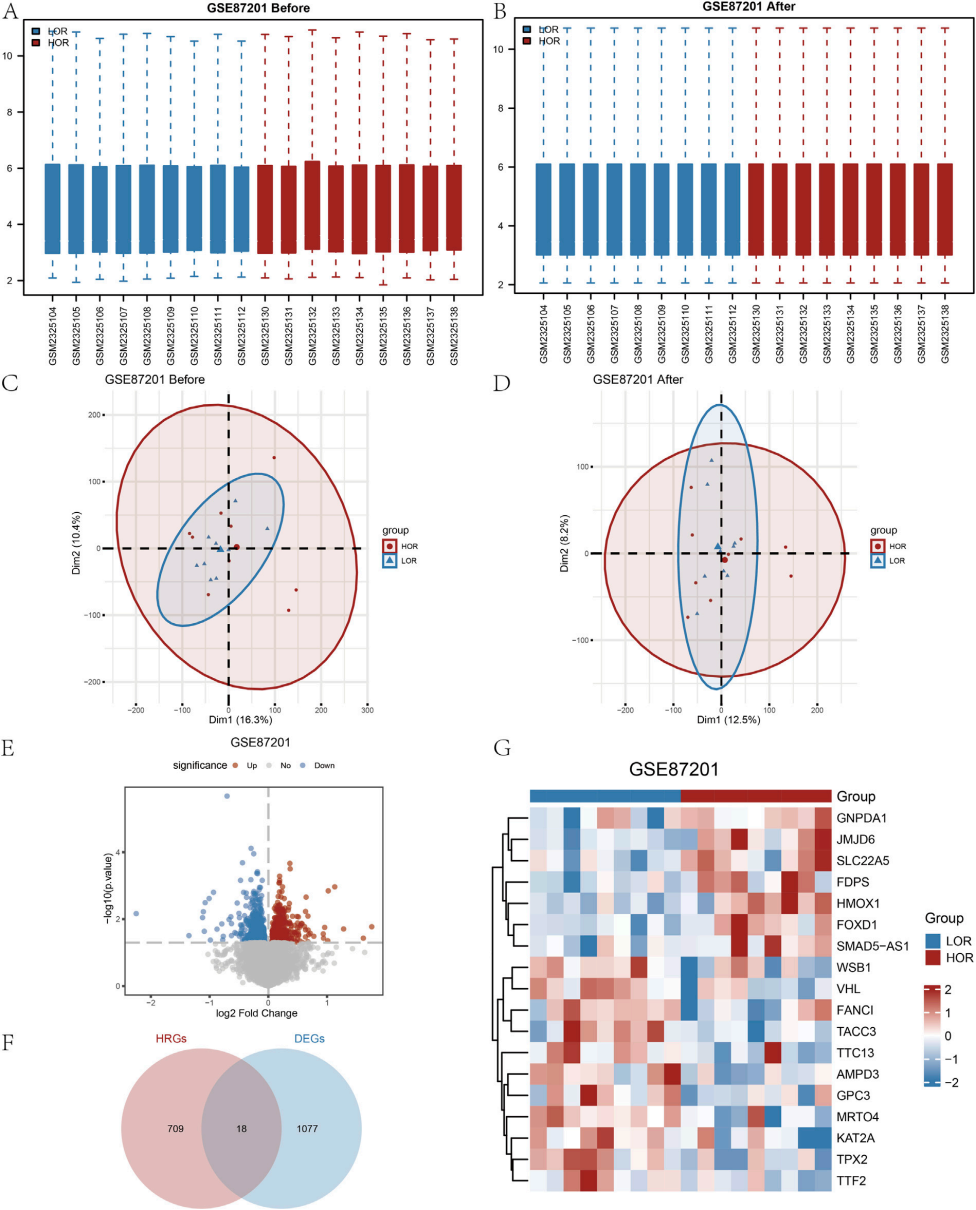
Processing of the diminished ovarian reserve (DOR) dataset and identification of the HRDEGs. **(A** and **B)** Before and after normalization of the GSE87201 dataset. **(C** and **D)** Before and after the PCA plots of GSE87201 removal of batch effect treatments. **(E)** Volcano plot of DEGs analyzed between LOR and HOR. **(F)** Venn plot of DEGs and HRGs in the dataset. **(G)** Complex numerical heatmap of HRDEGs in the GSE87201 dataset. DOR, diminished ovarian reserve; HRDEG, hypoxia-related differentially expressed gene; PCA, principal component analysis; DEG, differentially expressed gene; HRG, hypoxia relevant gene.

The DOR GSE87201 dataset underwent normalization utilizing the R package ‘limma.’ Following the removal of intersample batch effects, nine HOR and nine LOR samples were retained (Figures 2A,B). Validation of the expression matrix through PCA indicated that the batch effect was substantially mitigated after ComBat correction (Figures 2C,D). Specifically, Figure 2C shows batch-driven clustering before correction, while Figure 2D shows the dispersion of samples no longer follows batch grouping, reflecting effective batch adjustment. A total of 28,136 DEGs were identified, with 1,095 genes exhibiting a |logFC| greater than 0 and a *p*-value less than 0.05. Among these, 618 genes were upregulated, showing higher expression in LOR (and lower expression in HOR with LogFC > 0), while 477 genes were downregulated, exhibiting lower expression in LOR (and higher expression in HOR with LogFC < 0), as illustrated in the volcano plot (Figure 2E).

Based on the intersection of HRGs with Co-DEGs, we identified the following genes: *AMPD3*, *FANCI*, *FDPS*, *FOXD1*, *GNPDA1*, *GPC3*, *HMOX1*, *JMJD6*, *KAT2A*, *MRTO4*, *SLC22A5*, *SMAD5-AS1*, *TACC3*, *TPX2*, *TTC13*, *TTF2*, *VHL*, and *WSB1*. In total, 18 HRDEGs were depicted in a Venn diagram (Figure 2F).

From the heatmap (Figure 2G), it can be observed that there are differences in the expression patterns of various genes between the LOR and HOR groups, which may be related to ovarian function and egg quality. For instance, some genes such as *GNPDA1*, *JMJD6*, *SLC22A5*, *FDPS*, *HMOX1*, and *FOXD1* exhibit higher expression in the HOR group, which may be associated with better ovarian reserve and egg quality. Conversely, other genes such as *WSB1*, *VHL*, *FANCI*, *TACC3*, *TTC13*, *AMPD3*, *GPC3*, *MRTO4*, *KAT2A*, *TPX2*, and *TTF2* show higher expression in the LOR group, potentially correlating with reduced ovarian reserve and decreased egg quality (Figure 2G).

### 3.2 DOR dataset for GSEA and GSVA

**FIGURE 3:**
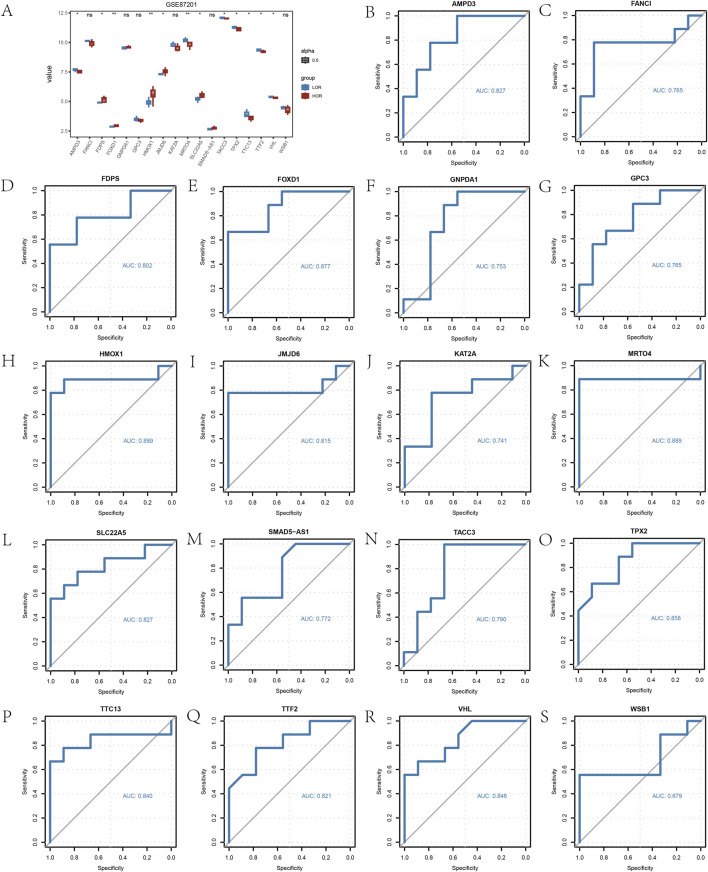
Gene set enrichment analysis (GSEA) of the GSE87201 dataset. **(A)** The main five biological characteristics of GSEA in the GSE87201 dataset. **(B–F)** The enriched signaling pathways. GSEA, gene set enrichment analysis.

**FIGURE 4:**
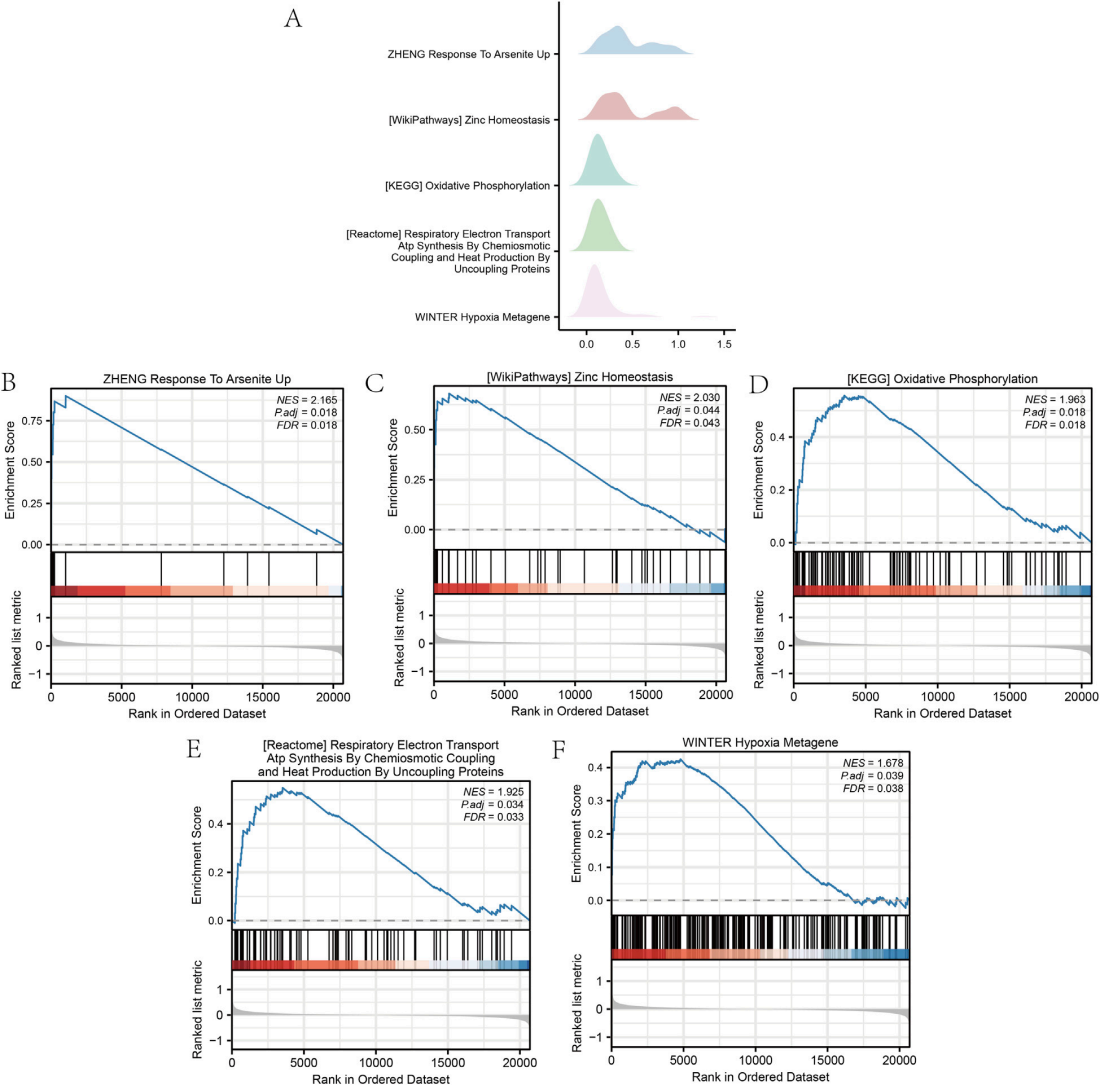
Gene set variation analysis (GSVA) of the DOR GSE87201 dataset. **(A)** Heatmap of the GSVA of genes in the LOR and HOR groups. **(B)** Group comparison plot of the analyzed results based on the Mann–Whitney *U* test. *p < 0.05; **p < 0.01; and ***p < 0.001. GSVA, gene set variation analysis; DOR, diminished ovarian reserve; LOR, low ovarian reserve; HOR, high ovarian reserve; ns, not statistically significant.

The GSEA of all genes from the GSE87201 dataset, utilizing the FDR *p*-value (q-value <0.25 and adjusted *p*-value < 0.05) as the criterion for enrichment screening, identified significant enrichment of DEGs in five biological processes: response to arsenite uptake (Pan et al., 2020; Chen et al., 2022; Eslami et al., 2024; Adeogun et al., 2025), zinc homeostasis, oxidative phosphorylation (Verma et al., 2018; Nasiadek et al., 2020; Camp et al., 2023; Liao et al., 2023; Liu et al., 2024), respiratory electron transport coupled with adenosine triphosphate synthesis (Anderson et al., 2018; Bao et al., 2024; Kobayashi et al., 2024), and hypoxia metagene (Lim et al., 2021; Huang et al., 2023; Gutzeit et al., 2024) (Figure 3). The GSVA identified 11 gene set pathways exhibiting distinct expression differences between the low and high outcome risk (LOR and HOR) groups, which were visualized using a heat map generated with the R package ‘pheatmap’ (Figure 4A). A comparative analysis of the degree of difference across these 11 pathways, conducted using the Mann–Whitney U test, indicated that eight pathways were statistically significant (*p* < 0.05) (Figure 4B). Detailed information on the five biological processes and the 11 gene set pathways is provided in Supplementary Tables S3 and S4.

### 3.3 Constructing ROC curves for HRDEGs

**FIGURE 5:**
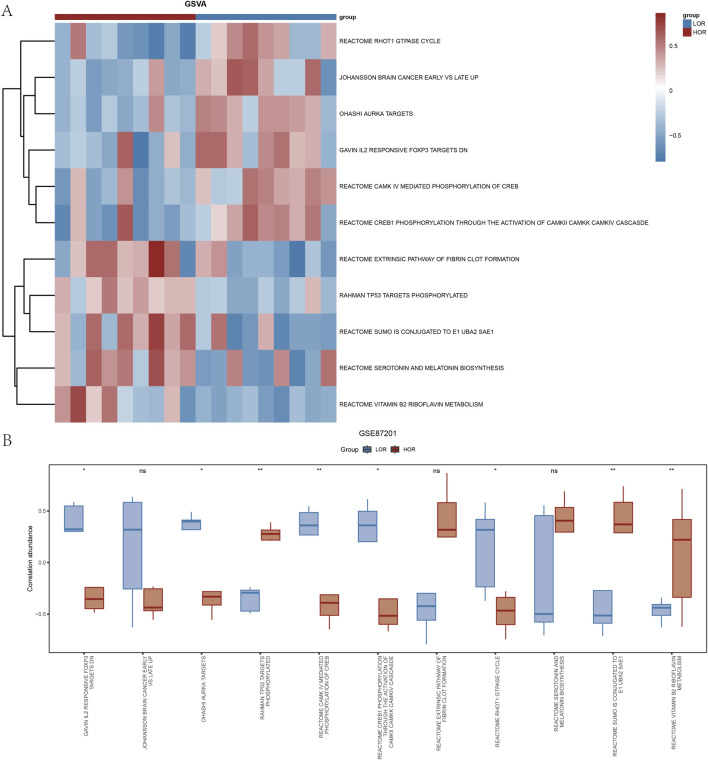
Differential analysis of hypoxia-related differentially expressed gene (HRDEGs) expression. **(A)** Group comparison plot of the analyzed results from HRDEGs. **(B)** ROC models for 18 HRDEGs. *p < 0.05; **p < 0.01; and ***p < 0.001. An AUC of 0.5–0.7 indicates low accuracy; an AUC of 0.7–0.9 indicates some accuracy; an AUC >0.9 indicates high accuracy.HRDEG, hypoxia-related differentially expressed gene; ROC, receiver operating characteristic; AUC, area under the curve; ns, not statistically significant.

Furthermore, the analysis of expression differences in 18 HRDEGs between the LOR and HOR groups, performed using the Wilcoxon signed-rank test, revealed that 12 HRDEGs were statistically significant (Figure 5A). ROC curves and AUC values for the 18 HRDEGs within the GSE87201 dataset were plotted individually (Figure 5B). The findings indicated a significant correlation between 17 HRDEGs and DOR.

### 3.4 Construction of the PPI and mRNA regulatory network

**FIGURE 6:**
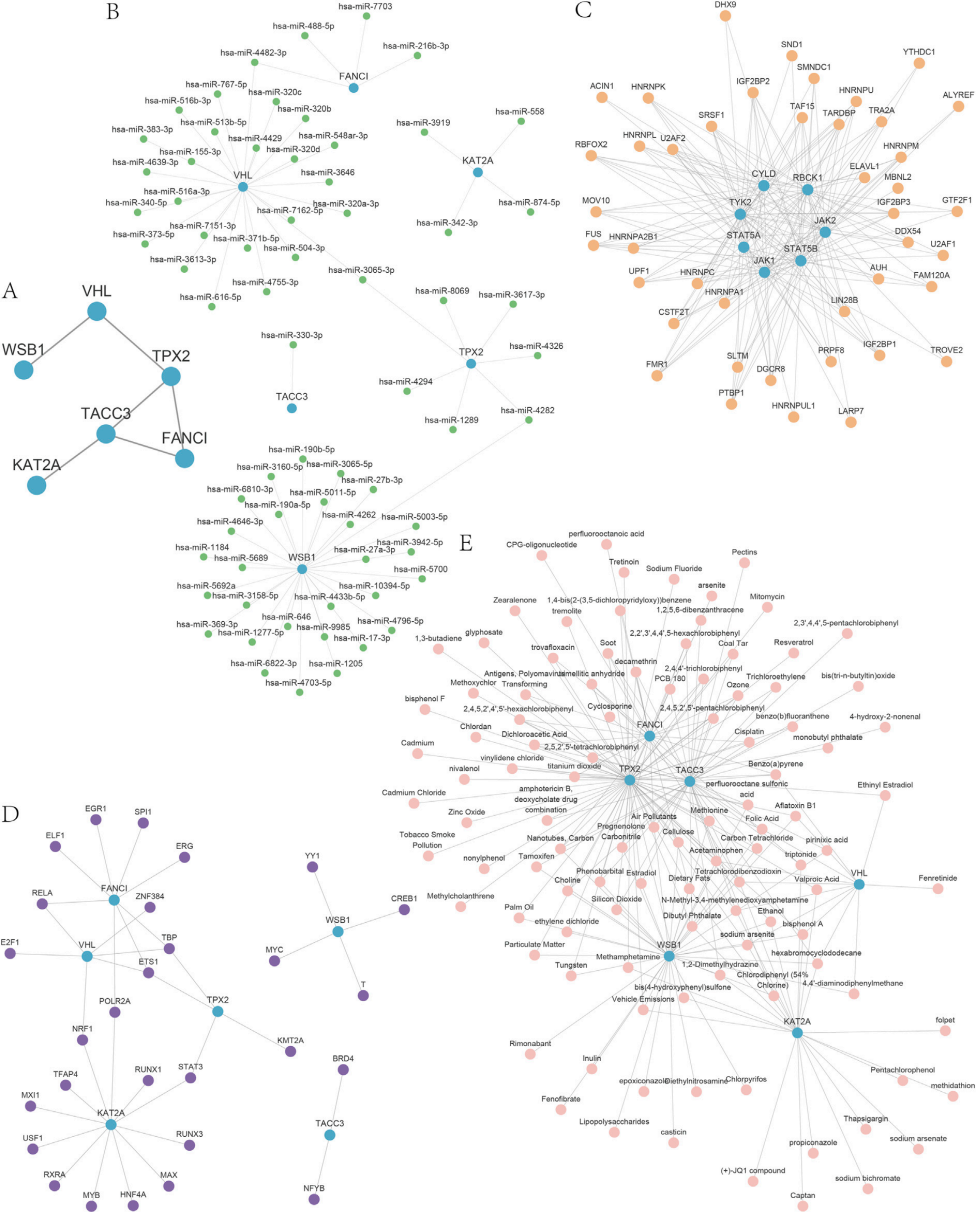
The PPI and mRNA regulatory network. **(A)** The PPI for HRDEGs. **(B)** mRNA–miRNA regulatory network of hub genes. **(C)** The mRNA–RBP network of hub genes. **(D)** The mRNA–TF network of hub genes. **(E)** The mRNA–drugs network of hub genes. Blue indicates the hub genes; green indicates miRNA; orange indicates RBP, purple indicates TF, and pink indicates a drug. PPI, protein–protein interaction; mRNA, messenger ribonucleic acid; HRDEG, hypoxia-related differentially expressed gene; miRNA, micro-ribonucleic acid; RBP, ribonucleic acid binding protein; TF, transcription factor.

To further elucidate the underlying mechanisms of hypoxia-related markers in DOR, we performed a protein-protein interaction (PPI) analysis on 18 HRDEGs within the DOR dataset, utilizing the STRING database with a medium confidence threshold of 0.400. The resultant data were imported into Cytoscape software to generate an interrelationship network diagram, which identified six hub genes: *FANCI*, *KAT2A*, *TACC3*, *TPX2*, *VHL*, and *WSB1* (Figure 6A).

The miRNAs interacting with these hub genes were predicted using the miRDB database, leading to the construction of an mRNA-miRNA regulatory network comprising 67 miRNA molecules and 70 mRNA-miRNA interaction pairs, as detailed in Supplementary Table S5 and depicted in Figure 6B. Additionally, the interactions between hub genes and RNA-binding proteins (RBPs) were predicted using the ENCORI database, revealing an mRNA–RBP network consisting of 23 RBP molecules and 77 mRNA-RBP interaction pairs (Figure 6C), with TPX2 interacting with 17 RBP molecules, as shown in Supplementary Table S6. Transcription factors binding to the hub genes were identified through the CHIPBase (version 2.0) and hTFtarget databases. The intersection facilitated the construction of a visualization network comprising 28 transcription factors and hub genes (Figure 6D), with *KAT2A* exhibiting 12 interaction pairs with transcription factors (Supplementary Table S7). Subsequently, the CTD database was utilized to predict 111 potential drugs or molecular compounds targeting the hub genes (Figure 6E), among which 72 were identified as targeting *TPX2* (Supplementary Table S8).

Collectively, these multi-omics analyses delineate a comprehensive regulatory landscape for the six hub genes, encompassing post-transcriptional control (miRNAs), RNA processing (RBPs), transcriptional regulation, and druggable targets. Notably, TPX2 emerges as the most interconnected node with both RBPs (17 partners) and therapeutic compounds (72 agents), highlighting its central role in DOR pathogenesis and potential as a priority target for intervention.

### 3.5 GO and KEGG analysis of hub genes

**FIGURE 7:**
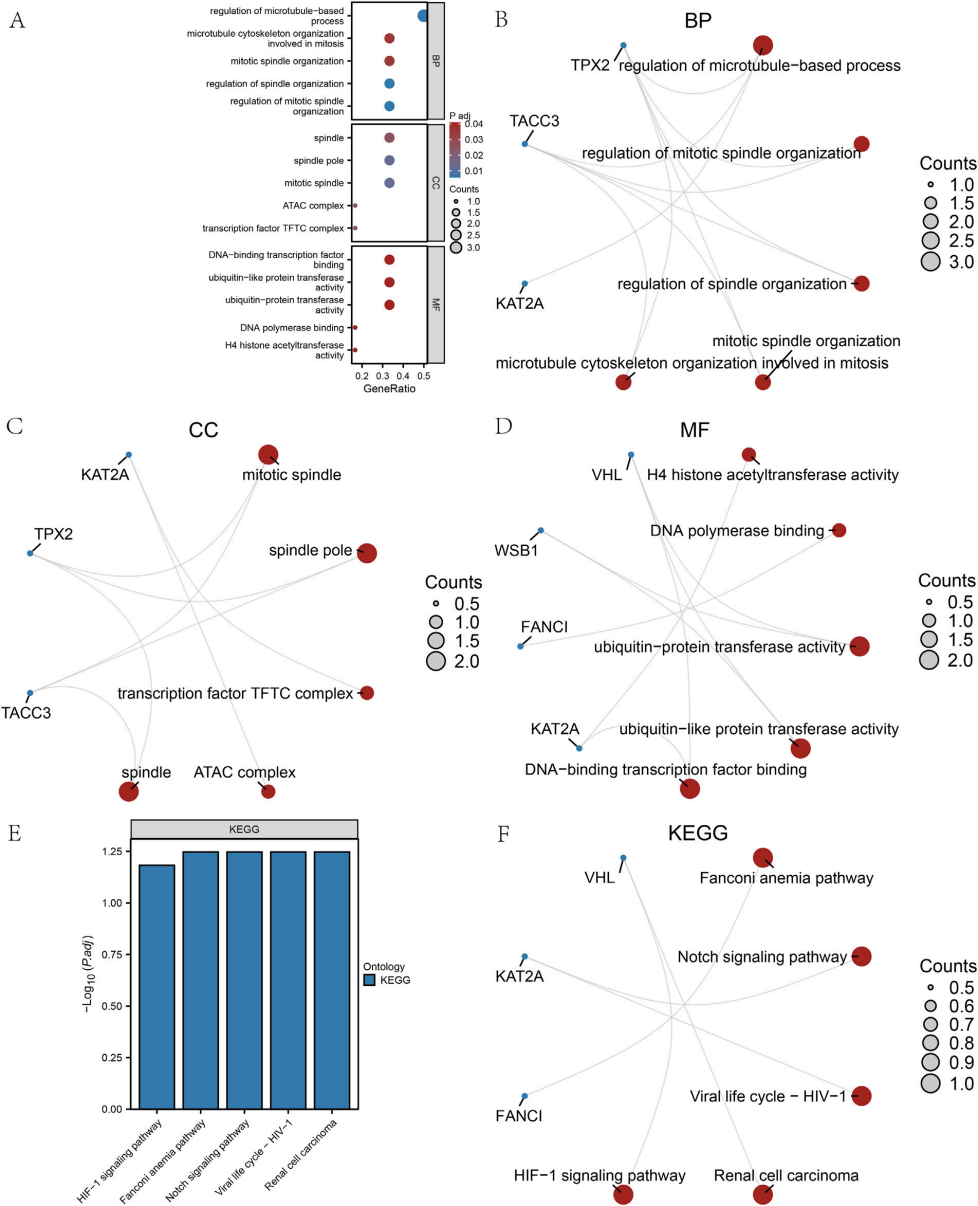
Gene ontology (GO) and Kyoto Encyclopedia of Genes and Genomes (KEGG) analyses of the hub genes. **(A)** The bubble diagram of GO analysis of the hub genes. **(B)** The biological process results for hub genes in the GO analysis. **(C)** The cell composition results for hub genes in the GO analysis. **(D)** The molecular function results for hub genes in the GO analysis. **(E)** Bar graph of KEGG analysis results. **(F)** Network graph of the KEGG analysis results. GO, gene ontology; KEGG, Kyoto Encyclopedia of Genes and Genomes; BP, biological process; CC, cell composition; MF, molecular function.

GO analysis, employing a screening threshold of *p* < 0.1 (Lu et al., 2004; Katsel et al., 2005; Alam-Faruque et al., 2014) and an FDR <0.05, revealed the enrichment of hub genes in DOR (Figure 7A). This includes genes associated with the regulation of microtubule-based processes, mitotic spindle organization, and microtubule cytoskeleton organization during mitosis, as well as other biological processes (Figure 7B). Additionally, the analysis identified cellular components (CCs) such as the mitotic spindle, spindle pole, transcription factor target coactivator complex, Ada2a-containing complex, and spindle cellular components (Figure 7C). Furthermore, MFs such as H4 histone acetyltransferase activity, DNA polymerase binding, ubiquitin-protein transferase activity, ubiquitin-like protein transferase activity, and DNA-binding transcription factor binding were enriched (Figure 7D).

The KEGG analysis revealed significant enrichment of hub genes within five specific pathways: the Fanconi anemia pathway, Notch signaling pathway, viral life cycle-HIV-1, renal cell carcinoma, and HIF-1 signaling pathway. These findings are illustrated through bar graphs (Figure 7E) and network graphs (Figure 7F). Detailed results of the GO and KEGG analyses can be found in Supplementary Tables S9 and S10.

### 3.6 Study of immune cell infiltration

**FIGURE 8:**
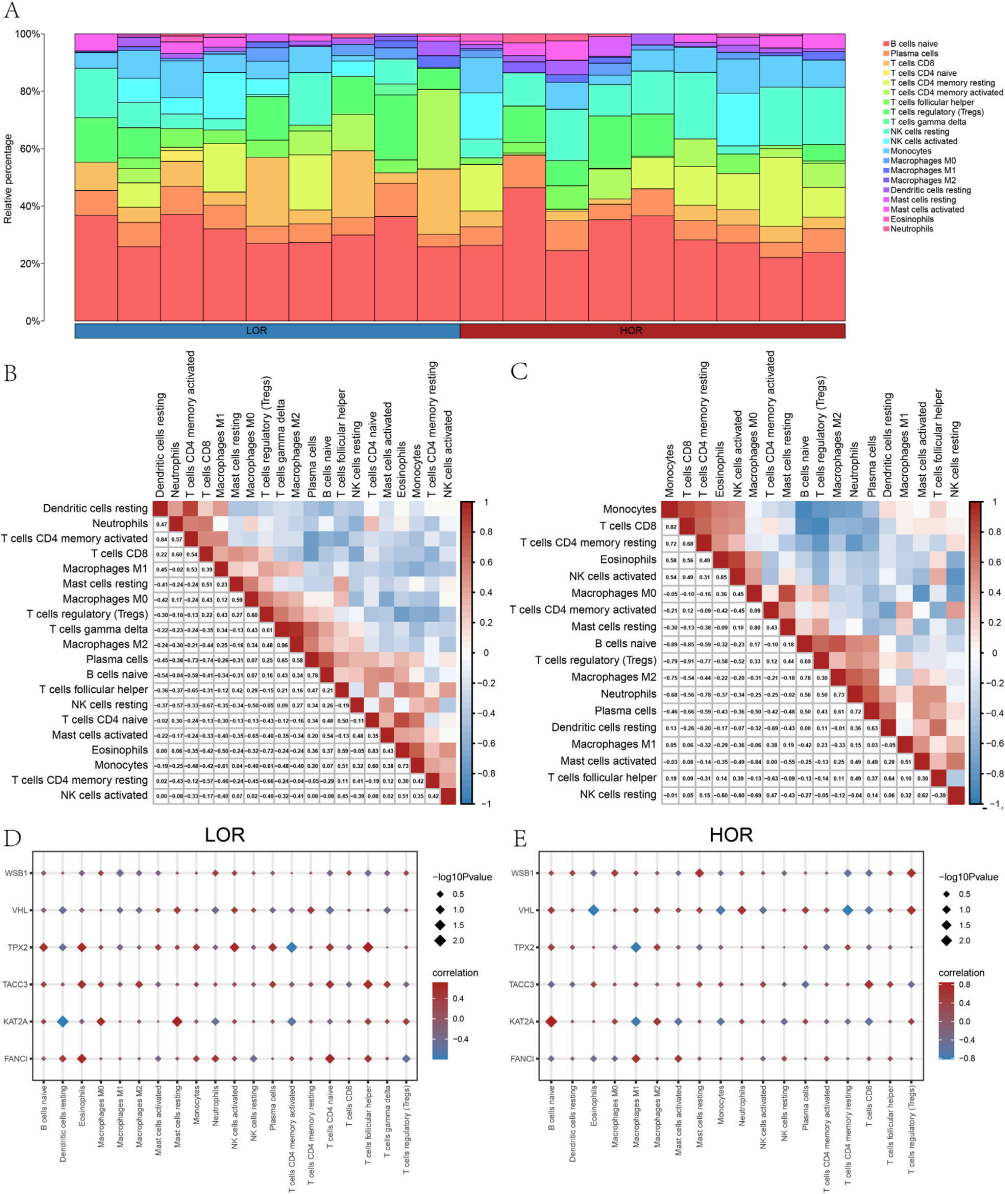
Analysis of immune cell infiltration. **(A)** Histogram of immune infiltration for the GSE87201 dataset. **(B)** Heatmap of the abundance of immune cell infiltration in low ovarian reserve (LOR). **(C)** Heatmap of the abundance of immune cell infiltration in high ovarian reserve (HOR). **(D)** Immunoassay plot of the hub genes in LOR. **(E)** Immunoassay plot of the hub genes in HOR. LOR, low ovarian reserve; HOR, high ovarian reserve.

Additionally, we employed the CIBERSORT package alongside the Pearson algorithm to assess the correlation of 22 immune cell types with the DOR GSE87201 dataset (Figures 8A–C) and to examine the relationship between hub genes and 20 immune cell types in LOR and HOR. In LOR, the strongest positive correlation was observed between M2 macrophages and gamma delta-T cells, while the strongest negative correlation was found between plasma cells and CD8 T cells. Notably, *FANCI* and *TACC3* exhibited significant positive associations with eosinophils, naïve CD4 T cells, and follicular helper T cells. Conversely, *KAT2A* showed a significant negative correlation with resting dendritic cells. Furthermore, CD4 T memory cells were significantly negatively correlated with resting dendritic cells and activated CD4 memory T cells, while demonstrating a positive correlation with follicular helper T cells. *TPX2* exhibited a positive association with eosinophils, naïve CD4 T cells, and follicular helper T cells, while demonstrating a negative correlation with activated CD4 memory T cells (Figure 8D). Within the HOR group, WSBI showed a positive correlation with resting mast cells and regulatory T cells. Conversely, *VHL* was negatively associated with eosinophils and resting CD4 T memory cells, yet positively correlated with regulatory T cells. *KAT2A* expression was positively correlated with naïve B cells and negatively correlated with M1 macrophages. Additionally, *TPX2* expression was negatively correlated with M1 macrophages (Figure 8E). In summary, *FANCI*, *TACC3*, and *TPX2* were significantly associated with various immune cells. These robust correlations delineate distinct immune microenvironment patterns associated with key hub genes in both LOR and HOR contexts. Notably, TPX2 consistently demonstrated significant associations across both groups, particularly with T cell subsets and eosinophils in LOR and M1 macrophages in HOR, suggesting its potentially broader role.

### 3.7 *In vitro* validation of the role of FANCI and KAT2A in DOR

**FIGURE 9:**
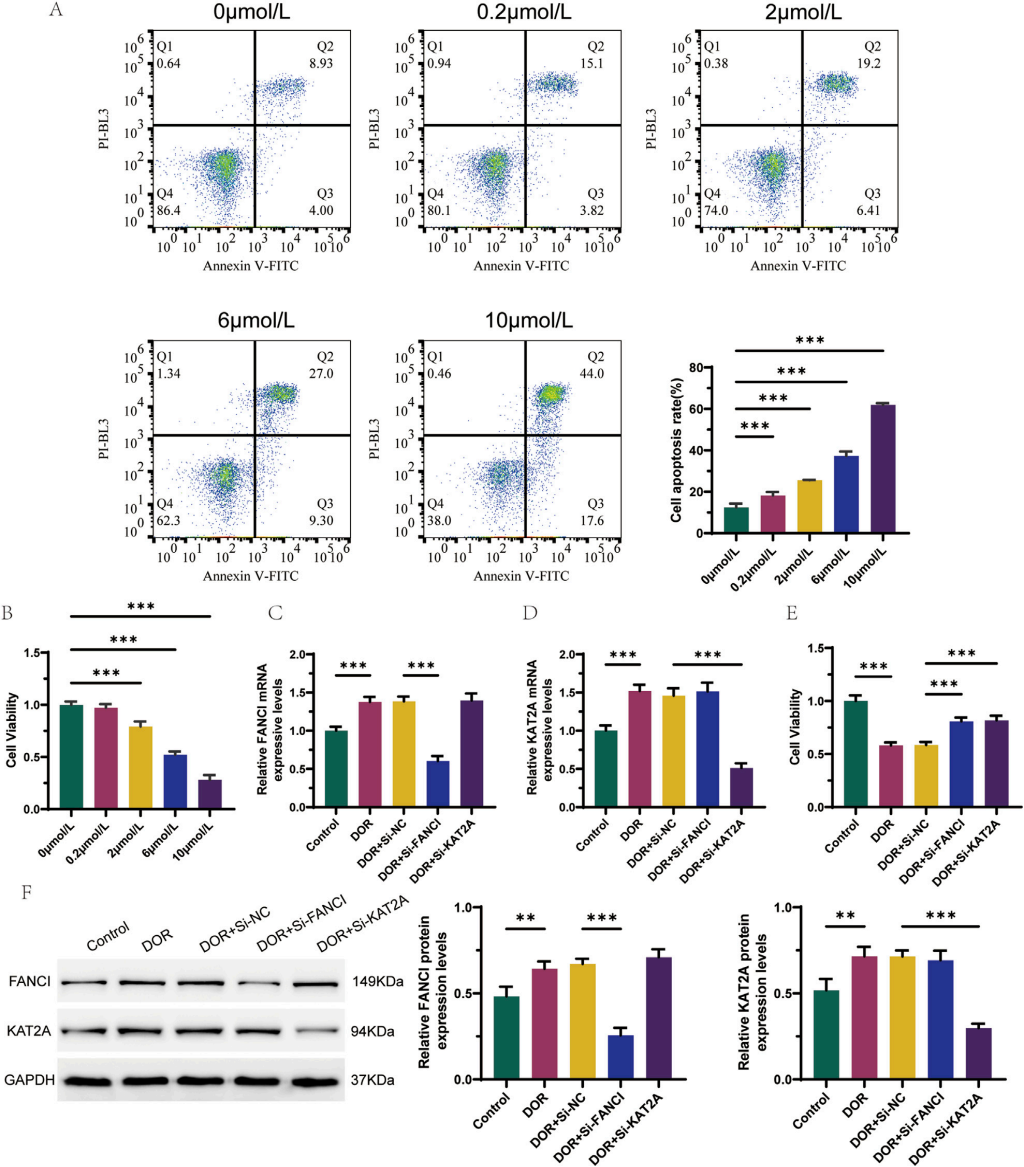
*In vitro* validation of the role of *FANCI* and *KAT2A* in DOR. **(A)** Flow cytometry detection of apoptosis for optimal concentration of 4-HC induced granulocyte injury models. **(B)** CCK8 activity test screens for optimal concentration of 4-HC induced granulocyte injury models. **(C)** Differences in the expression levels of *FANCI* were detected by RT-qPCR. **(D)** Differences in the expression levels of *KAT2A* were detected by RT-qPCR. **(E)** Cell viability in different groups by CCK8 assay. **(F)** Differences in protein expression levels of *FANCI* and *KAT2A* were detected by Western blot. *p < 0.05; **p < 0.01; and ***p < 0.001.

To validate the results of the bioinformatics analysis, a 4-HC-induced granulosa cell injury model was employed. Among the six identified hub genes, *FANCI* and *KAT2A* were randomly selected for preliminary validation to provide a representative functional assessment. Flow cytometry analysis revealed that the apoptosis rate of KGN cells increased with higher drug concentrations, while cell viability decreased, as determined by the CCK8 activity assay (Figures 9A,B). Compared to the Control group, the mRNA expression levels of *FANCI* and *KAT2A* were significantly elevated in the DOR group. In contrast, the mRNA expression level of *FANCI* was significantly reduced in the DOR + Si-FANCI group, and the mRNA expression level of *KAT2A* was significantly reduced in the DOR + Si-KAT2A group, relative to the DOR + Si-NC group (Figures 9C,D). These findings were corroborated at the protein level (Figure 9F). Furthermore, cell viability in the DOR group was significantly lower compared to the Control group, whereas cell viability in the DOR + Si-FANCI and DOR + Si-KAT2A groups was significantly higher compared to the DOR + Si-NC group (Figure 9E).

**FIGURE 10:**
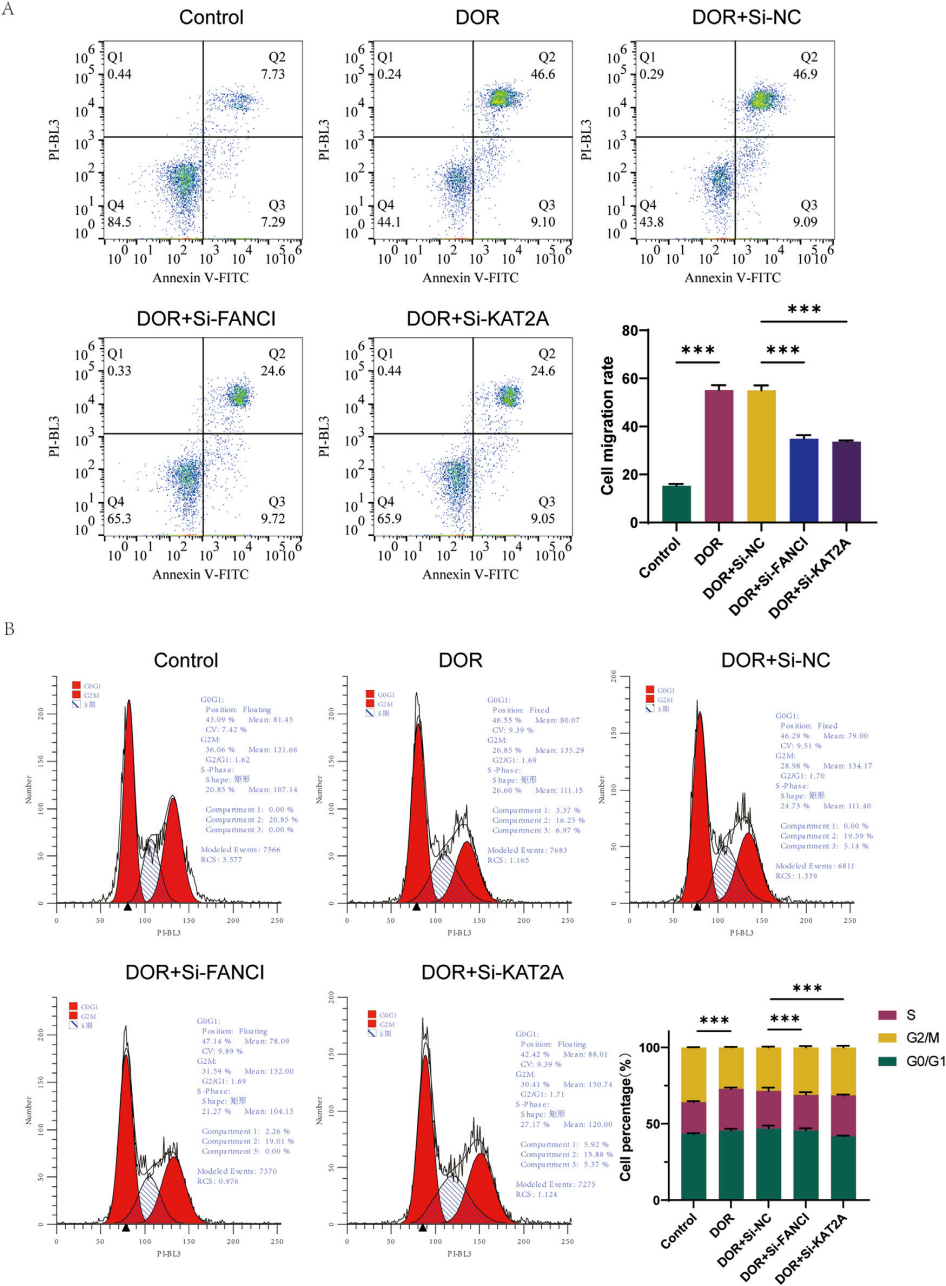
The role of FANCI and KAT2A in DOR by low assay. **(A)** Flow cytometry detection of apoptosis. **(B)** Cell cycle test by flow assay. *p < 0.05; **p < 0.01; and ***p< 0.001.

Furthermore, the percentage of apoptosis was significantly elevated in the DOR group compared to the Control group, and significantly reduced in the DOR + Si-FANCI and DOR + Si-KAT2A groups compared to the DOR + Si-NC group (Figure 10A). In the DOR group, there was a significant increase in the number of cells in the G0/G1 phase and a significant decrease in the number of cells in the S/G2 phase compared to the Control group. Conversely, in the DOR + Si-NC group, the number of cells in the G0/G1 phase significantly decreased, while the number of cells in the S/G2 phase significantly increased compared to the DOR + Si-FANCI and DOR + Si-KAT2A groups (Figure 10B).

Collectively, these rigorous *in vitro* functional assays robustly validate our bioinformatic predictions, demonstrating that both FANCI and KAT2A are functionally implicated in granulosa cell injury associated with DOR. Silencing either gene effectively mitigated the detrimental effects of 4-HC, as evidenced by significantly reduced apoptosis, restored cell viability, and normalized cell cycle progression (reduced G0/G1 arrest and increased S/G2 phase cells) compared to the non-targeting siRNA control in the injury model. These findings solidify FANCI and KAT2A as critical drivers of granulosa cell dysfunction in DOR and underscore their potential as therapeutic targets for mitigating ovarian damage.

## 4 Discussion

This study aimed to analyze DEGs in patients with DOR. We identified six hub genes—*FANCI*, *KAT2A*, *TACC3*, *TPX2*, *VHL*, and *WSB1*—that may serve as diagnostic biomarkers for DOR. These findings could facilitate the development of novel therapeutic strategies targeting hypoxia-induced DOR. Local tissue hypoxia has been implicated in the induction of programmed cell death in ovarian granulosa cells, thereby diminishing ovarian reserve function and ultimately impairing female fertility. However, sensitive and specific biomarkers characterizing the local hypoxic environment have not yet been clearly identified. Recent studies have underscored a strong association between hypoxia-induced apoptosis of ovarian granulosa cells and genetic alterations (Tao et al., 2021).

In this study, we initially examined 1,095 DEGs in LOR compared to HOR using the GSE87201 dataset, identifying 618 upregulated and 477 downregulated genes. Subsequent analysis identified 18 HRDEGs. Employing the Wilcoxon signed-rank test, we developed a ROC risk model, which highlighted six hub genes: *FANCI*, *KAT2A*, *TACC3*, *TPX2*, *VHL*, and *WSB1*. We then constructed mRNA interaction networks involving miRNAs, RNA-binding proteins, transcription factors, and potential therapeutic compounds. Our findings indicated that *TPX2* interacted with 17 RBP molecules, *KAT2A* was involved in 12 mRNA-TF interaction pairs, and 72 drugs or compounds targeted *TPX2*.

*FANCI* is a critical component of the Fanconi anemia pathway, a classical DNA damage repair mechanism. The FA pathway is primarily responsible for repairing DNA interstrand cross-links and maintaining replication fork stability, thereby playing a significant role in ovarian development and function (Nalepa and Clapp, 2018). Research has demonstrated that *FANCI* knockout mice display aberrant primordial germ cell cycles, diminished proliferation, and inadequate folliculogenesis, which ultimately results in the premature depletion of follicular reserves (Dubois et al., 2019). Although *KAT2A*, a histone acetyltransferase, has not been directly associated with DOR (Wang et al., 2017), studies suggest that elevated levels of *KAT2A* can stabilize hypoxia-inducible factor 1-alpha (HIF-1α), thereby fostering a localized hypoxic environment (Wang et al., 2020). Prior research has demonstrated that *KAT2A* is integral to the activation of the NLRP3 inflammasome (Zhang et al., 2023; Zhu et al., 2024), which subsequently results in the accumulation of succinate (Zhu et al., 2024). This accumulation can, through multiple mechanisms, activate the HIF-1αsignaling pathway, thereby influencing cellular metabolism and inflammatory responses (Xu et al., 2025). *TACC3*, a motor spindle protein, plays a crucial role in stabilizing spindles during mitosis (Mahdipour et al., 2015). Mutations in *TACC3* have been linked to defective spindle assembly and impaired oocyte growth (Wu W. et al., 2022). Additionally, *TPX2*, a spindle-associated protein, is integral to spindle orientation and assembly (Mosler et al., 2022). The inhibition of *TPX2* during oocyte maturation can lead to abnormal spindle morphology or instability, resulting in cell apoptosis (Goshima, 2011; Ding et al., 2019). A key function of *VHL* is to facilitate the degradation of HIF-1α through the ubiquitin-proteasome pathway (Tarade and Ohh, 2018). The inactivation of the *VHL* gene facilitates the accumulation of HIF-1α, which subsequently promotes the expression of cytokines such as vascular endothelial growth factor and transforming growth factor-α. This process accelerates cellular energy metabolism, vascular growth, and other related processes (Perrotta et al., 2020). HIF-1α may confer protection to granulosa cells against apoptosis by mitigating oxidative stress and altering the Bax/Bcl-2 balance, thereby enhancing ovarian reserve function (Tang et al., 2021). *WSB1* proteins, which are downstream hypoxia-inducible proteins regulated by HIF-1α, play a role in modulating the degradation of various proteins involved in the cellular response to hypoxia (Haque et al., 2016). Given the alignment between our findings and previous research, we hypothesize that *FANCI*, *TACC3*, and *TPX2* are closely associated with DOR, whereas *KAT2A*, *VHL*, and *WSB1* may participate in the cellular mechanisms underlying hypoxia.

GO and KEGG analyses indicated that the hub genes implicated in DOR are predominantly enriched in the regulation of microtubule-based processes, mitotic spindle organization, and the organization of the microtubule cytoskeleton involved in mitosis. Furthermore, these genes were found to be enriched in several key biological pathways, including the Fanconi Anemia pathway, the Notch signaling pathway, the viral life cycle of HIV-1, renal cell carcinoma, and the hypoxia-inducible factor 1 (HIF-1) signaling pathway. The FA pathway is a well-established DNA damage repair mechanism that plays a crucial role in the repair of interstrand cross-links and the maintenance of replication fork stability (Vanni et al., 2022). Its primary components include damage recognition, recruitment of the FA core complex, monosialization of the FANCD2–FANCI complex, and subsequent downstream processes such as nucleolytic cleavage, translesion synthesis, and homologous recombination (Zhao et al., 2023). Deficiencies in multiple FA genes can adversely affect primordial germ cell development and oocyte meiosis, resulting in abnormal follicular development and a reduced ovarian reserve (Kupfer, 2013).

The Notch signaling pathway, a highly conserved evolutionary intracellular pathway, is integral to oogenesis, ovarian hormone secretion, and the regulation of germline stem cell proliferation and differentiation (Guo et al., 2021). Notch1, Hes1, and Hes5 serve as critical receptors and downstream target molecules within the Notch pathway, with their expression levels indicative of the pathway’s activity (Vanorny et al., 2014). The transition from mitosis to internal replication during the differentiation of follicular cells is modulated by the Notch signaling pathway. Inhibition of this pathway has been shown to prevent mouse oocytes from progressing into meiosis (Patiño et al., 2019). Hes1 plays a critical role in the formation and recruitment of primordial follicular cisterns; oocyte development is significantly impaired in Hes1-knockout mice, resulting in a marked reduction in oocyte numbers (Manosalva et al., 2013). HIF-1α has emerged as a significant pathway in recent years, promoting granulosa cell autophagy and apoptosis through the induction of pro-apoptotic proteins and the modulation of various signaling pathways, thereby contributing to DOR (Jia et al., 2020). Research indicates that the accumulation of HIF-1α in the cytoplasm under hypoxic conditions leads to its overexpression in follicular granulosa cells. Furthermore, HIF-1α binds to hypoxia-responsive elements within the promoter region of *BNIP3*, resulting in the upregulation of *BNIP3* and subsequent promotion of granulosa cell apoptosis (Yuan et al., 2018). In addition, HIF-1α interacts with HIF-1β within the nucleus to form a dimer, which subsequently enhances the expression of the target gene *BNIP3*. This gene competes with Beclin-1, a crucial component in the autophagy process, for binding to Bcl-2, leading to the dissociation of Beclin-1 from Bcl-2 and the induction of autophagy in granulosa cells (Abrahamsen et al., 2012). These findings align with our research, which demonstrated the involvement of FA, Notch, and HIF-1α signaling pathways in the mechanism underlying hypoxic DOR.

Increasing evidence suggests that hypoxia is a critical driver of ovarian dysfunction, acting through mechanisms that include oxidative stress, mitochondrial impairment, and inflammation. In the context of DOR, chronic hypoxic stress may disrupt follicular homeostasis by modulating gene expression, immune surveillance, and granulosa cell viability. Among the post-transcriptional regulators identified in this study, HNRNPK has been shown to modulate ROS homeostasis and cellular aging under hypoxic conditions *via* HIF-1α-dependent signaling pathways (Razorenova, 2012; Naarmann-de Vries et al., 2016; Chen et al., 2019). Similarly, SRSF1 contributes to cell migration and angiogenesis by regulating the alternative splicing of HIF-1α, thereby integrating hypoxic signaling with vascular remodeling (Chang and Lin, 2019). These findings highlight a mechanistic link between hypoxia and DOR, wherein transcriptional and post-transcriptional modulators may converge to disrupt ovarian microenvironmental integrity.

Critically, our study reveals that *FANCI*, *TACC3*, and *TPX2* expression positively correlates with eosinophil infiltration in LOR samples. Eosinophils exhibit variable distribution within the ovary (Standaert et al., 1991), demonstrating a rapid increase in response to luteinizing hormone (Masciangelo et al., 2021), followed by a significant decline in the absence of estrogen. These cells play a crucial role in the immune response by presenting antigens to T lymphocytes. Their recruitment is influenced by the synergistic effects of cytokines, adhesion molecules, and chemokines (Suzuki et al., 2003), which release mediators that combat inflammation and disease. This association suggests that hypoxia-related hub genes may amplify inflammatory responses within hypoxic follicles through eosinophil-mediated antigen presentation and tissue remodeling, potentially exacerbating granulosa cell apoptosis in DOR pathology.

Through the integration of differential immune infiltration analysis and its subsequent correlation, we discerned a positive association between M2 macrophages and gamma-delta T cells, as well as a significant negative correlation between plasma cells and CD8 T cells in the context of DOR. Further investigation into the expression of hub genes within immune cells in the DOR setting revealed that *FANCI*, *TACC3*, and *TPX2* were upregulated in eosinophils, naïve CD4 T cells, and follicular helper T cells. Conversely, *KAT2A* and *TPX2* were downregulated in activated CD4 memory T cells. Notably, *KAT2A* also showed differential expression in resting dendritic cells, further implicating it in immune-endocrine crosstalk within the ovarian microenvironment. In addition, our mRNA–drug interaction network analysis (Figure 6E) identified *TPX2* as a promising therapeutic target, with 72 candidate compounds predicted to interact with it, indicating translational potential for drug development in hypoxia-associated ovarian dysfunction. The observed correlations between key hypoxia-related hub genes and specific immune cell populations suggest that these genes may influence ovarian immunoregulation and contribute to follicular dysfunction under stress.

T follicular helper (Tfh) cells, a specialized subset of CD4^+^ naïve T cells, secrete interleukin-21 during immune stabilization *in vivo*. This secretion facilitates the proliferation and activation of B cells, as well as the transformation of immune proteins, subsequently impacting the secretion and metabolism of sex hormones in patients. Such mechanisms may significantly contribute to the etiology of DOR (Pernis, 2007; Crotty, 2011). Notably, we observed co-regulation of *FANCI*, *TACC3*, and *TPX2* with Tfh cells, implicating these genes in disrupted immune-endocrine crosstalk. Given that estrogen deficiency in DOR impairs CD4^+^ T cell function *via* ERα/ERβ, hypoxia-driven dysregulation of Tfh activity may further compromise follicular development through altered sex hormone metabolism. A study (Priyanka et al., 2013) demonstrated that estrogen modulates CD4^+^ T cell function through its interaction with nuclear receptors, specifically estrogen receptor alpha (ERα) and estrogen receptor beta (ERβ), located on CD4^+^ T cells. Zhang et al. observed that the increased infiltration of selective T lymphocytes in mice resulted in a reduction of CD4^+^ T cells and an augmentation of CD8^+^ T cells within ovarian tissues, thus influencing the CD4+/CD8+ T cell ratio. CD4^+^ memory cells represent a minor subset of differentiated CD4^+^ T cells that facilitate a renewed immune response (Miyahara et al., 2008). In a separate investigation (Wu et al., 2007), it was found that CD45RO, a subpopulation of CD4 memory T cells, exhibited a similar distribution of follicular cells within both its HOR and LOR groups. Kryczek et al. reported that memory T cells contribute to follicular degeneration, leading to DOR, by delivering cytotoxic signals that induce apoptosis in oocytes or supportive granulosa cells (Kryczek et al., 2005).

Dendritic cells (DCs), recognized as the most effective antigen-presenting cells due to their extensive repertoire of immunorecognitive receptors, play a crucial role in the intrinsic immune response by secreting substantial quantities of cytokines (Steinman, 2012). In recent years, dendritic cells have been identified in follicular cells, where they facilitate the activation of effector T cells through the secretion of inflammatory cytokines such as interleukin-6 (IL-6) and interleukin-23 (IL-23) (Fainaru et al., 2012). Our findings further indicate that *KAT2A* exhibits a negative correlation with resting dendritic cells. Given that *KAT2A* plays a role in stabilizing HIF-1ɑ, this observation suggests that hypoxic conditions may inhibit DC-mediated T cell activation. This is consistent with the observed reduction in interleukin-23 (IL-23) and elevation in interleukin-10 (IL-10) levels within DOR follicular fluid. Therefore, the dysfunction of DCs driven by *KAT2A* likely contributes to the acceleration of follicular atresia through the disruption of immunomodulatory pathways. In patients exhibiting impaired ovarian function, there is a noted decrease in the inflammatory cytokine IL-23 within follicular fluid, alongside an increase in the concentration of the anti-inflammatory cytokine IL-10. This cytokine imbalance results in a reduced number of DCs, which are consequently less effective in activating T cells. Such a scenario may contribute to follicular developmental disorders and a subsequent decline in ovarian function (Duan et al., 2014).

Collectively, the correlations between hypoxia-related hub genes (*FANCI*, *KAT2A*, *TACC3*, *TPX2*) and immune cells (eosinophils, Tfh, DCs) reveal their synergistic roles in ovarian immunopathology. These interactions position hypoxia-responsive genes as pivotal regulators of the immune landscape in DOR, modulating inflammatory microenvironments, immune-endocrine crosstalk, and follicular integrity. Although the mechanisms by which HRDEGs influence the immunomodulation of DOR remain underexplored, it is hypothesized—based on prior studies (Silva et al., 2014; Huang et al., 2017) and the findings of the current study—that HRDEGs may play a significant role in the immunopermeability associated with DOR. To further validate the functional relevance of *FANCI* and *KAT2A* in DOR pathogenesis, we conducted *in vitro* experiments using a 4-HC–induced granulosa cell injury model. Hypoxia induces DNA damage and apoptosis *via* ROS accumulation, and 4-HC effectively simulates this process, rendering the model pathologically comparable. Although 4-HC is not a direct hypoxic agent, its mechanism of inducing oxidative stress through ROS accumulation closely parallels the downstream cellular consequences of hypoxia, including mitochondrial dysfunction, HIF-1α stabilization, and apoptosis. Previous studies have demonstrated that hypoxia-induced injury in granulosa cells involves oxidative damage pathways that are similarly activated by 4-HC (Zhu et al., 2022). Thus, the 4-HC-induced granulosa injury model serves as a relevant and practical surrogate for evaluating hypoxia-related pathogenic mechanisms in DOR. Hypoxia induces DNA damage and apoptosis *via* ROS accumulation, and 4-HC effectively simulates this process, rendering the model pathologically comparable. Consistent with our transcriptomic findings, the mRNA expression levels of FANCI and KAT2A were significantly upregulated in the DOR group compared to controls. Functionally, silencing either gene (Si-FANCI or Si-KAT2A) led to a marked increase in cell viability relative to the DOR + Si-NC group, suggesting a pro-apoptotic role of these genes under oxidative stress. Flow cytometry analysis revealed that cell apoptosis rates were significantly elevated in the DOR model but were substantially reduced following silencing of FANCI or KAT2A. Moreover, cell cycle analysis demonstrated that 4-HC treatment induced G0/G1 phase arrest, while knockdown of either gene partially rescued the cell cycle progression into S and G2 phases. These findings suggest that *FANCI* and *KAT2A* may promote granulosa cell apoptosis and impair proliferation by regulating both apoptosis and cell cycle checkpoints, further providing evidence to support *in silico* results.

This study has several limitations. First, although efforts were made to correct for batch effects, the use of publicly available transcriptomic datasets may have introduced unavoidable batch-to-batch variability. Second, while six hub genes were identified through bioinformatics analysis, only two (*FANCI* and *KAT2A*) were selected for experimental validation. This selection was made through a randomization strategy to avoid bias, but due to time and resource constraints, the remaining four hub genes (*TACC3*, *TPX2*, *VHL*, and *WSB1*) could not be validated during this study cycle. These genes will be prioritized in future work involving both *in vitro* experiments and clinical sample analyses. Third, the lack of clinical correlation data limited our ability to directly link gene expression patterns to patient phenotypes or hormonal profiles. Integrating clinical validation with mechanistic studies will be a key focus of our future research. Fourth, the absence of hypoxia induction experiments (e.g., CoCl_2_ treatment) and rescue assays (e.g., HIF-1α knockdown) limits the ability to establish direct causal relationships between hypoxic stress and the regulation of hub genes. Future research might aim to conduct hypoxia induction and pathway-specific rescue experiments to validate the regulatory roles of these hub genes and further elucidate their mechanistic involvement in hypoxia-associated DOR pathogenesis.

## 5 Conclusion

In summary, we identified six hub genes associated with hypoxia and DOR, namely, *FANCI*, *KAT2A*, *TACC3*, *TPX2*, *VHL*, and *WSB1*. These genes were validated in the context of immune infiltration. The findings provide valuable insights for further elucidating the pathogenesis of hypoxia-induced decline in ovarian reserve function. Nonetheless, the specific pathogenesis and molecular targets of hypoxia-associated DOR warrant further investigation.

## Data Availability

The raw data supporting the conclusions of this article will be made available by the authors, without undue reservation.

## Abbreviations

DOR, Diminished ovarian reserve; Co-DEGs, Common differentially expressed genes; HRDEGs; Hypoxia-related differentially expressed genes; ROC, Receiver operating characteristic curve; GO, Gene ontology; KEGG, Kyoto Encyclopedia of Genes and Genomes; HOR, High ovarian reserve; LOR, Low ovarian reserve; GEO database, Gene Expression Omnibus database; GSEA, Gene set enrichment analysis; FDR, False discovery rate; MF, Molecular function; AUC, Area under the curve; PPI, Protein–protein interaction; STRING, Search Tool for the Retrieval of Interacting Genes; miRNA, Micro-ribonucleic acid; mRNA, Messenger ribonucleic acid; RBP, RNA binding protein; TF, Transcription factor; CTD, Comparative toxicogenomics database; CC, Cellular component; HIF-1α, Hypoxia-inducible factor 1-alpha.
