# Supplementary material for: Identification of hypoxia-related diagnostic biomarkers and immune signatures in diminished ovarian reserve
Source: Front Genet. 2025 Aug 4;16:1626992. doi: 10.3389/fgene.2025.1626992 (PMC12358289; doi:10.3389/fgene.2025.1626992)
Supplement: Supplementary file 1 [file Table1.docx]

**Table 1. List of Diminished ovarian reserve datasets information.**

|  | GSE87201 |
| --- | --- |
| Platform | GPL17586 |
| Type | Expression profiling by array |
| Species | Homo sapiens |
| Tissue | Oocytes |
| Samples in LOR group | 9 |
| Samples in HOR group | 9 |
| Reference | The transcriptome of human oocytes is related to age and ovarian reserve. |

LOR：Low ovarian reserve；HOR：High ovarian reserve。
