# Supplementary material for: Identification of hypoxia-related diagnostic biomarkers and immune signatures in diminished ovarian reserve
Source: Front Genet. 2025 Aug 4;16:1626992. doi: 10.3389/fgene.2025.1626992 (PMC12358289; doi:10.3389/fgene.2025.1626992)
Supplement: Supplementary file 2 [file Table8.docx]

**Table S4. mRNA-drugs interaction network nodes.**

| mRNA | drug | mRNA | drug |
| --- | --- | --- | --- |
| FANCI | 1,4-bis(2-(3,5-dichloropyridyloxy))benzene | TPX2 | Aflatoxin B1 |
| FANCI | 2,2',3',4,4',5-hexachlorobiphenyl | TPX2 | Air Pollutants |
| FANCI | 2,4,4'-trichlorobiphenyl | TPX2 | amphotericin B, deoxycholate drug combination |
| FANCI | 2,4,5,2',4',5'-hexachlorobiphenyl | TPX2 | Antigens, Polyomavirus Transforming |
| FANCI | 2,4,5,2',5'-pentachlorobiphenyl | TPX2 | arsenite |
| FANCI | 2,5,2',5'-tetrachlorobiphenyl | TPX2 | Benzo(a)pyrene |
| FANCI | Air Pollutants | TPX2 | benzo(b)fluoranthene |
| FANCI | Antigens, Polyomavirus Transforming | TPX2 | bis(4-hydroxyphenyl)sulfone |
| FANCI | Benzo(a)pyrene | TPX2 | bisphenol A |
| FANCI | Cisplatin | TPX2 | bisphenol F |
| FANCI | CPG-oligonucleotide | TPX2 | Cadmium |
| FANCI | decamethrin | TPX2 | Cadmium Chloride |
| FANCI | Dichloroacetic Acid | TPX2 | Cellulose |
| FANCI | Dietary Fats | TPX2 | Chlordan |
| FANCI | Nanotubes, Carbon | TPX2 | Choline |
| FANCI | Ozone | TPX2 | Cisplatin |
| FANCI | PCB 180 | TPX2 | Coal Tar |
| FANCI | perfluorooctanoic acid | TPX2 | decamethrin |
| FANCI | Pregnenolone Carbonitrile | TPX2 | Dibutyl Phthalate |
| FANCI | Soot | TPX2 | Dichloroacetic Acid |
| FANCI | Tetrachlorodibenzodioxin | TPX2 | Dietary Fats |
| FANCI | titanium dioxide | TPX2 | Estradiol |
| FANCI | trimellitic anhydride | TPX2 | Ethanol |
| FANCI | triptonide | TPX2 | Folic Acid |
| FANCI | trovafloxacin | TPX2 | glyphosate |
| KAT2A | 1,2-Dimethylhydrazine | TPX2 | Methamphetamine |
| KAT2A | bis(4-hydroxyphenyl)sulfone | TPX2 | Methionine |
| KAT2A | bisphenol A | TPX2 | Methoxychlor |
| KAT2A | Captan | TPX2 | Methylcholanthrene |
| KAT2A | Dibutyl Phthalate | TPX2 | Mitomycin |
| KAT2A | Dietary Fats | TPX2 | monobutyl phthalate |
| KAT2A | Ethanol | TPX2 | Nanotubes, Carbon |
| KAT2A | folpet | TPX2 | nivalenol |
| KAT2A | hexabromocyclododecane | TPX2 | N-Methyl-3,4-methylenedioxyamphetamine |
| KAT2A | (+)-JQ1 compound | TPX2 | nonylphenol |
| KAT2A | Methamphetamine | TPX2 | Ozone |
| KAT2A | methidathion | TPX2 | Palm Oil |
| KAT2A | N-Methyl-3,4-methylenedioxyamphetamine | TPX2 | Particulate Matter |
| KAT2A | Pentachlorophenol | TPX2 | PCB 180 |
| KAT2A | propiconazole | TPX2 | perfluorooctane sulfonic acid |
| KAT2A | sodium arsenate | TPX2 | Phenobarbital |
| KAT2A | sodium arsenite | TPX2 | Pregnenolone Carbonitrile |
| KAT2A | sodium bichromate | TPX2 | Resveratrol |
| KAT2A | Tetrachlorodibenzodioxin | TPX2 | Silicon Dioxide |
| KAT2A | Thapsigargin | TPX2 | sodium arsenite |
| KAT2A | triptonide | TPX2 | Sodium Fluoride |
| KAT2A | Valproic Acid | TPX2 | titanium dioxide |
| KAT2A | Vehicle Emissions | TPX2 | Tobacco Smoke Pollution |
| TACC3 | Benzo(a)pyrene | TPX2 | tremolite |
| TACC3 | Carbon Tetrachloride | TPX2 | Tretinoin |
| TACC3 | pirinixic acid | TPX2 | Trichloroethylene |
| TACC3 | bis(tri-n-butyltin)oxide | TPX2 | trimellitic anhydride |
| TACC3 | Phenobarbital | TPX2 | trovafloxacin |
| TACC3 | Soot | TPX2 | Tungsten |
| TACC3 | 1,2,5,6-dibenzanthracene | TPX2 | Valproic Acid |
| TACC3 | 1,2-Dimethylhydrazine | TPX2 | Vehicle Emissions |
| TACC3 | 2,2',3',4,4',5-hexachlorobiphenyl | TPX2 | vinylidene chloride |
| TACC3 | 2,3',4,4',5-pentachlorobiphenyl | TPX2 | Zearalenone |
| TACC3 | 2,4,4'-trichlorobiphenyl | TPX2 | Zinc Oxide |
| TACC3 | 2,4,5,2',4',5'-hexachlorobiphenyl | VHL | 4,4'-diaminodiphenylmethane |
| TACC3 | 2,4,5,2',5'-pentachlorobiphenyl | VHL | Acetaminophen |
| TACC3 | 2,5,2',5'-tetrachlorobiphenyl | VHL | Aflatoxin B1 |
| TACC3 | 4-hydroxy-2-nonenal | VHL | Benzo(a)pyrene |
| TACC3 | Acetaminophen | VHL | bisphenol A |
| TACC3 | Air Pollutants | VHL | Ethinyl Estradiol |
| TACC3 | amphotericin B, deoxycholate drug combination | VHL | Fenretinide |
| TACC3 | benzo(b)fluoranthene | VHL | Folic Acid |
| TACC3 | Chlorodiphenyl (54% Chlorine) | VHL | hexabromocyclododecane |
| TACC3 | Choline | VHL | N-Methyl-3,4-methylenedioxyamphetamine |
| TACC3 | Cisplatin | VHL | sodium arsenite |
| TACC3 | Coal Tar | VHL | Tetrachlorodibenzodioxin |
| TACC3 | Cyclosporine | WSB1 | bisphenol A |
| TACC3 | decamethrin | WSB1 | Carbon Tetrachloride |
| TACC3 | Dibutyl Phthalate | WSB1 | Dietary Fats |
| TACC3 | Dichloroacetic Acid | WSB1 | ethylene dichloride |
| TACC3 | Estradiol | WSB1 | pirinixic acid |
| TACC3 | Ethinyl Estradiol | WSB1 | 1,2-Dimethylhydrazine |
| TACC3 | Folic Acid | WSB1 | 4,4'-diaminodiphenylmethane |
| TACC3 | hexabromocyclododecane | WSB1 | Acetaminophen |
| TACC3 | Methionine | WSB1 | Aflatoxin B1 |
| TACC3 | Nanotubes, Carbon | WSB1 | Air Pollutants |
| TACC3 | N-Methyl-3,4-methylenedioxyamphetamine | WSB1 | casticin |
| TACC3 | Ozone | WSB1 | Cellulose |
| TACC3 | PCB 180 | WSB1 | Chlorodiphenyl (54% Chlorine) |
| TACC3 | Pectins | WSB1 | Chlorpyrifos |
| TACC3 | perfluorooctane sulfonic acid | WSB1 | Choline |
| TACC3 | Pregnenolone Carbonitrile | WSB1 | Diethylnitrosamine |
| TACC3 | Silicon Dioxide | WSB1 | epoxiconazole |
| TACC3 | Tamoxifen | WSB1 | Estradiol |
| TACC3 | Tetrachlorodibenzodioxin | WSB1 | Ethanol |
| TACC3 | titanium dioxide | WSB1 | Fenofibrate |
| TACC3 | Trichloroethylene | WSB1 | Folic Acid |
| TACC3 | trimellitic anhydride | WSB1 | hexabromocyclododecane |
| TACC3 | triptonide | WSB1 | Inulin |
| TACC3 | vinylidene chloride | WSB1 | Lipopolysaccharides |
| TPX2 | Carbon Tetrachloride | WSB1 | Methionine |
| TPX2 | ethylene dichloride | WSB1 | Nanotubes, Carbon |
| TPX2 | Soot | WSB1 | Palm Oil |
| TPX2 | Tetrachlorodibenzodioxin | WSB1 | Particulate Matter |
| TPX2 | 1,2,5,6-dibenzanthracene | WSB1 | perfluorooctane sulfonic acid |
| TPX2 | 1,3-butadiene | WSB1 | Phenobarbital |
| TPX2 | 1,4-bis(2-(3,5-dichloropyridyloxy))benzene | WSB1 | Pregnenolone Carbonitrile |
| TPX2 | 2,2',3',4,4',5-hexachlorobiphenyl | WSB1 | Rimonabant |
| TPX2 | 2,4,4'-trichlorobiphenyl | WSB1 | Silicon Dioxide |
| TPX2 | 2,4,5,2',4',5'-hexachlorobiphenyl | WSB1 | sodium arsenite |
| TPX2 | 2,4,5,2',5'-pentachlorobiphenyl | WSB1 | Tamoxifen |
| TPX2 | 2,5,2',5'-tetrachlorobiphenyl | WSB1 | Tungsten |
| TPX2 | Acetaminophen | WSB1 | Vehicle Emissions |

“mRNA”and“drug”represent node；“-”represent edge
