# Supplementary material for: Identification of hypoxia-related diagnostic biomarkers and immune signatures in diminished ovarian reserve
Source: Front Genet. 2025 Aug 4;16:1626992. doi: 10.3389/fgene.2025.1626992 (PMC12358289; doi:10.3389/fgene.2025.1626992)
Supplement: Supplementary file 3 [file Table7.docx]

**Table S3. mRNA-TF interaction network nodes.**

| mRNA | TF | mRNA | TF |
| --- | --- | --- | --- |
| FANCI | EGR1 | KAT2A | TFAP4 |
| FANCI | ELF1 | KAT2A | USF1 |
| FANCI | ERG | TACC3 | BRD4 |
| FANCI | ETS1 | TACC3 | NFYB |
| FANCI | POLR2A | TPX2 | ETS1 |
| FANCI | RELA | TPX2 | KMT2A |
| FANCI | SPI1 | TPX2 | STAT3 |
| FANCI | TBP | TPX2 | TBP |
| KAT2A | HNF4A | VHL | E2F1 |
| KAT2A | MAX | VHL | ETS1 |
| KAT2A | MXI1 | VHL | NRF1 |
| KAT2A | MYB | VHL | RELA |
| KAT2A | NRF1 | VHL | TBP |
| KAT2A | POLR2A | VHL | ZNF384 |
| KAT2A | RUNX1 | WSB1 | CREB1 |
| KAT2A | RUNX3 | WSB1 | MYC |
| KAT2A | RXRA | WSB1 | T |
| KAT2A | STAT3 | WSB1 | YY1 |

“mRNA”and“TF”represent node；“-”represent edge；TF：Transcription factors.
