# Supplementary material for: Identification of hypoxia-related diagnostic biomarkers and immune signatures in diminished ovarian reserve
Source: Front Genet. 2025 Aug 4;16:1626992. doi: 10.3389/fgene.2025.1626992 (PMC12358289; doi:10.3389/fgene.2025.1626992)
Supplement: Supplementary file 4 [file Table9.docx]

**Table 5. GO enrichment analysis results of hub genes .**

| Ontology | ID | Description | GeneRatio | BgRatio | pvalue | p.adjust |
| --- | --- | --- | --- | --- | --- | --- |
| BP | GO:0032886 | regulation of microtubule-based process | 3/6 | 249/18800 | 4.46e-05 | 0.0057 |
| BP | GO:0060236 | regulation of mitotic spindle organization | 2/6 | 39/18800 | 6.26e-05 | 0.0057 |
| BP | GO:0090224 | regulation of spindle organization | 2/6 | 43/18800 | 7.62e-05 | 0.0057 |
| BP | GO:0007052 | mitotic spindle organization | 2/6 | 124/18800 | 0.0006 | 0.0354 |
| BP | GO:1902850 | microtubule cytoskeleton organization involved in mitosis | 2/6 | 151/18800 | 0.0009 | 0.0354 |
| CC | GO:0072686 | mitotic spindle | 2/6 | 160/19594 | 0.0010 | 0.0125 |
| CC | GO:0000922 | spindle pole | 2/6 | 169/19594 | 0.0011 | 0.0125 |
| CC | GO:0033276 | transcription factor TFTC complex | 1/6 | 14/19594 | 0.0043 | 0.0246 |
| CC | GO:0140672 | ATAC complex | 1/6 | 14/19594 | 0.0043 | 0.0246 |
| CC | GO:0005819 | spindle | 2/6 | 402/19594 | 0.0060 | 0.0246 |
| MF | GO:0010485 | H4 histone acetyltransferase activity | 1/6 | 12/18410 | 0.0039 | 0.0411 |
| MF | GO:0070182 | DNA polymerase binding | 1/6 | 22/18410 | 0.0071 | 0.0411 |
| MF | GO:0004842 | ubiquitin-protein transferase activity | 2/6 | 433/18410 | 0.0078 | 0.0411 |
| MF | GO:0019787 | ubiquitin-like protein transferase activity | 2/6 | 458/18410 | 0.0087 | 0.0411 |
| MF | GO:0140297 | DNA-binding transcription factor binding | 2/6 | 470/18410 | 0.0091 | 0.0411 |

GO：Gene Ontology；BP：Biological process；CC：Cellular component；MF：Molecular function。
