# Supplementary material for: Identification of hypoxia-related diagnostic biomarkers and immune signatures in diminished ovarian reserve
Source: Front Genet. 2025 Aug 4;16:1626992. doi: 10.3389/fgene.2025.1626992 (PMC12358289; doi:10.3389/fgene.2025.1626992)
Supplement: Supplementary file 5 [file Table2.docx]

**Table 2. Hypoxia related genes related genes list.**

| Hypoxia related genes | | | | | |
| --- | --- | --- | --- | --- | --- |
| A4GALT | PGM2 | IER3 | HIGD1AP9 | DDX41 | TES |
| ABCC4 | PHF14 | IGFBP1 | MIR210 | DEPDC1 | TFCP2 |
| ABHD12 | PHF19 | IGFBP3 | COL1A1 | DEPDC1B | TGFB3 |
| ACKR3 | PHF21A | ILF3 | NHIP | DHDH | TGFBI |
| ACLY | PHKG1 | ILVBL | EGLN1P1 | DHX9 | TGM2 |
| ACOT7 | PHLDA2 | INHA | EGLN3P1 | DKK1 | TIGD5 |
| ACTR5 | PIK3IP1 | INTS3 | ENSG00000254591 | DLGAP5 | TIMELESS |
| ACYP1 | PIM1 | IRS2 | ENSG00000270577 | DNAJB11 | TIPARP |
| ADCK2 | PIR | IRX3 | ENSG00000271291 | DNAJB6 | TIPIN |
| ADORA2B | PITX1 | ISG20 | ENSG00000271455 | DNAJC9 | TKTL1 |
| AHSA1 | PKLR | ITGA5 | ENSG00000271439 | DNMT1 | TLE6 |
| AK4 | PKP1 | ITGAE | TNF | DOLK | TMEM201 |
| AKAP12 | PKP4 | JMJD6 | SCARNA5 | DONSON | TMEM45A |
| AKR1B10 | PLAC8 | KAT2A | BDNF-AS | DPYSL4 | TMEM45B |
| ALDOA | PLAUR | KDELR3 | CASP9 | DSCC1 | TNFAIP3 |
| ALDOB | PLIN2 | KDM3A | MAPK1 | DTL | TNFRSF21 |
| ALDOC | PLK1 | KIAA0100 | SOD2-OT1 | DTNA | TOP2A |
| ALG6 | PNCK | KIF20A | CERNA3 | DUS1L | TOR3A |
| ALKBH2 | PNRC1 | KIF20B | CREBBP | DUSP1 | TPBG |
| AMPD3 | POLA2 | KIF23 | NOS1 | DUT | TPD52 |
| ANAPC4 | POLE2 | KIF2C | SMAD5-AS1 | E2F2 | TPI1 |
| ANGEL2 | POLG2 | KIF4A | MIR7-3HG | ECT2 | TPST2 |
| ANKZF1 | POLQ | KIF5A | AKT1 | EDN2 | TPX2 |
| ANXA2 | POLR2L | KLF6 | IL1B | EFNA1 | TROAP |
| APOO | POLR3K | KLF7 | TGFB1 | EFNA3 | TSEN2 |
| ARID3B | POP1 | KLHL24 | LINC01672 | EME1 | TSEN54 |
| ARV1 | PPARGC1A | LALBA | CXCL8 | ENO1 | TSPAN8 |
| ASNS | PPFIA4 | LARGE1 | EDN1 | ENO2 | TTC13 |
| ASPM | PPIH | LBR | NOS2 | ENO3 | TTF2 |
| ATF3 | PPP1R15A | LDHC | PTGS2 | EPDR1 | TTK |
| ATP5MC1 | PPP1R3C | LOX | TMX2-CTNND1 | ERO1A | TTL |
| ATP7A | PPP1R3E | LRP11 | CUL2 | ERRFI1 | TUBA1A |
| ATRIP | PRC1 | LRP8 | MYC | ESCO2 | TUBB6 |
| AURKA | PRDX5 | LSM4 | HSP90AA1 | ETS1 | TUFT1 |
| AURKB | PRICKLE4 | LXN | NOS3 | EXO1 | TUSC2 |
| B3GALT6 | PRIM1 | LYAR | TIGAR | EXOSC2 | TXN |
| B4GALNT2 | PRKCA | MAD2L1 | CYB5R3 | EXOSC8 | TYMS |
| BANF1 | PRR11 | MAFF | MAPK14 | EXT1 | TYSND1 |
| BCAN | PRR7 | MANEAL | BDNF | F3 | UBE2T |
| BCS1L | PSME3 | MAP3K1 | HIPK2 | FAM111A | UBL4A |
| BGN | PSPH | MCM2 | KDR | FAM111B | UCN2 |
| BHLHE40 | PUSL1 | MCM3 | NFKB1 | FAM13A | UGP2 |
| BIRC5 | PYGM | MCM4 | VEGFC | FAM72D | UHRF1 |
| BIRC7 | RACGAP1 | MCM5 | REST | FAM83D | USP1 |
| BLM | RAN | MCM6 | NFE2L2 | FANCB | USP39 |
| BNIP3L | RANGAP1 | MCM7 | FLT1 | FANCG | VEGF |
| BRI3BP | RBM15B | MCRS1 | ANGPT2 | FANCI | VLDLR |
| BRS3 | RBM45 | MCTS1 | RWDD3 | FASN | WDR12 |
| BTG1 | RBPJ | MED10 | LEP | FBP1 | WDR4 |
| BUB1B | RCC2 | MELK | MMP9 | FBXO5 | WDR54 |
| CA12 | RCN2 | METTL13 | NOTCH1 | FDPS | WDR92 |
| CA5B | RCOR2 | METTL3 | MMP2 | FEN1 | WFS1 |
| CACYBP | RDH13 | MIF | BAX | FERMT1 | WISP2 |
| CAD | REXO4 | MIIP | SDHB | FHL3 | WSB1 |
| CASP2 | RFC3 | MRPL24 | TH | FIBCD1 | XPNPEP1 |
| CASP6 | RFC4 | MRPL52 | STAT3 | FKBP14 | XPO1 |
| CATSPER1 | RFWD3 | MRPS17 | SP1 | FLVCR1 | XRCC3 |
| CAV1 | RFX5 | MRPS23 | MGARP | FN3KRP | YEATS2 |
| CAVIN1 | RHOQ | MRTO4 | EGR1 | FOS | ZMYM1 |
| CAVIN3 | RPIA | MSH2 | CCL2 | FOSL2 | ZMYND19 |
| CCDC138 | RPUSD1 | MT1E | SOD1 | FOXD1 | ZNF114 |
| CCDC14 | RRAGD | MT1X | MDM2 | FOXM1 | ZNF142 |
| CCN1 | RRAS | MT2A | ELOC | FOXO3 | ZNF165 |
| CCN5 | RRM1 | MTG1 | CXCL12 | FOXRED2 | ZNF232 |
| CCNA2 | RRM2 | MXI1 | PSMA7 | FSCN2 | ZNF292 |
| CCNB1 | RRP15 | MYH9 | EPOR | FTSJ3 | ZNF30 |
| CCNF | RRP7A | NAE1 | SRC | G6PD | ZNF416 |
| CCNG2 | RRS1 | NAGK | CAMK4 | GAA | ZNF503 |
| CCNO | RUVBL1 | NBEAL2 | PTEN | GADD45B | ZNF512B |
| CCT5 | S100A4 | NCAN | LOC107303340 | GALK1 | ZNF518A |
| CD3EAP | SAAL1 | NCAPD2 | SIRT1 | GALR2 | ZSCAN16 |
| CDC123 | SAC3D1 | NCAPG | SPP1 | GAPDH | ZWILCH |
| CDC20 | SAP30 | NCKIPSD | LRP5 | GAPDHS | HIF1A |
| CDC25A | SCARB1 | NCOA5 | FGF2 | GBE1 | EGLN1 |
| CDC25C | SDC2 | NDC80 | PWAR1 | GCK | HIF3A |
| CDC7 | SDC3 | NDST1 | RHOA | GCNT2 | HIF1AN |
| CDCA2 | SDC4 | NDST2 | MAPK8 | GINS2 | EGLN2 |
| CDCA3 | SDF2L1 | NEDD4L | NDP | GINS3 | EPAS1 |
| CDCA4 | SELENBP1 | NEDD8 | MIR21 | GINS4 | ARNT |
| CDCA7 | SETMAR | NEIL3 | PIK3CG | GLRX | HYOU1 |
| CDCA8 | SKA1 | NEK2 | PGR-AS1 | GMNN | HILPDA |
| CDK5 | SKA2 | NFIL3 | MAPK3 | GNB1L | SETD2 |
| CDK6 | SKP2 | NFXL1 | ALKBH5 | GNPDA1 | EPO |
| CDKN1A | SLBP | NHP2 | ELOB | GPC1 | HIGD1A |
| CDKN1B | SLC22A5 | NME1 | DELEC1 | GPC3 | CA9 |
| CDKN1C | SLC25A1 | NOCT | ACE | GPC4 | RAB4B-EGLN2 |
| CDKN3 | SLC25A19 | NOL11 | XDH | GPI | HIGD2A |
| CDT1 | SLC2A1 | NOL3 | THBS1 | GRHPR | HIGD1B |
| CENPA | SLC2A14 | NOP56 | INS | GTF3C2 | TP53 |
| CENPB | SLC2A3 | NQO1 | TLR4 | GYS1 | EP300 |
| CENPE | SLC2A5 | NR3C1 | TWIST1 | HABP4 | HIGD1C |
| CENPF | SLC37A4 | NUAK1 | IGF1 | HAS1 | HIGD2B |
| CENPI | SLC38A6 | NUF2 | ADAM17 | HASPIN | CASP3 |
| CENPK | SLC46A1 | NUP107 | SESN2 | HCFC1 | MTOR |
| CENPM | SLC5A6 | NUP155 | PVT1 | HDAC1 | BNIP3 |
| CENPO | SLC6A6 | NUP188 | ESR1 | HDAC4 | HIGD1AP1 |
| CEP55 | SLC6A8 | NUP37 | IFNG | HDLBP | H19 |
| CHAF1A | SLC7A11 | NUP62 | ADM | HELLS | P4HTM |
| CHAF1B | SLC7A6 | NUP85 | ANGPTL4 | HEXA | CTNNB1 |
| CHCHD3 | SMTN | NUPL2 | BCL2 | HIST2H2BE | CREB1 |
| CHEK1 | SNRPC | NXT2 | CCN2 | HK1 | HIGD1AP18 |
| CHRNA5 | SNRPD1 | P4HA1 | CITED2 | HLA-G | HIGD1AP11 |
| CHST10 | SNRPF | P4HA2 | CXCR4 | HMBS | HIGD1AP13 |
| CHST2 | SOX18 | PA2G4 | DDIT4 | HMMR | HIGD1AP16 |
| CHST3 | SPAG4 | PALLD | EGFR | HNRNPA1 | HIGD1AP5 |
| CIP2A | SPC25 | PAM | EGLN3 | HNRNPA3 | HIGD1AP10 |
| CKAP2 | SPIN4 | PAQR4 | FAM162A | HOXB9 | HIGD1AP12 |
| CKLF | SQLE | PARP1 | HK2 | HS3ST1 | HIGD1AP15 |
| CKS2 | SRPX | PBK | HMOX1 | HSPA14 | HIGD1AP2 |
| COL5A1 | STBD1 | PCK1 | IL6 | HSPA1A | HIGD1AP3 |
| COL7A1 | STC1 | PCNA | JUN | HSPA1B | HIGD1AP4 |
| COPS6 | STC2 | PCSK9 | LDHA | HSPA4 | HIGD1AP6 |
| CP | STRBP | PDGFB | NDRG1 | HSPA5 | HIGD1AP8 |
| CPSF6 | STX10 | PDK3 | PDK1 | HSPB1 | HIGD1AP14 |
| CRYBB2 | STXBP5 | PDSS1 | PFKFB4 | HSPH1 | HIGD1AP17 |
| CSE1L | STXBP6 | PFAS | PGF | IDS | HIGD1AP7 |
| CSRP2 | SULT2B1 | PFDN6 | RORA | DCLRE1B | TARBP1 |
| CSTF2 | TACC3 | PFKFB3 | SERPINE1 | DCN | TBC1D7 |
| DARS2 | TAF4 | PFKL | SIAH2 | DCTPP1 | TCHP |
| DCK | TAP2 | PFKP | SLC2A1 | DDIT3 | TCTEX1D2 |
| PGAM1 | VEGFA | PGAM2 | VHL | PGK1 | ZFP36 |
| PGM1 |  |  |  |  |  |
