# Supplementary material for: Identification of hypoxia-related diagnostic biomarkers and immune signatures in diminished ovarian reserve
Source: Front Genet. 2025 Aug 4;16:1626992. doi: 10.3389/fgene.2025.1626992 (PMC12358289; doi:10.3389/fgene.2025.1626992)
Supplement: Supplementary file 6 [file Table5.docx]

**Table S1. mRNA-miRNA interaction network nodes.**

| mRNA | miRNA | mRNA | miRNA |
| --- | --- | --- | --- |
| FANCI | hsa-miR-216b-3p | WSB1 | hsa-miR-5700 |
| FANCI | hsa-miR-7703 | WSB1 | hsa-miR-4262 |
| FANCI | hsa-miR-4482-3p | WSB1 | hsa-miR-646 |
| FANCI | hsa-miR-488-5p | WSB1 | hsa-miR-6810-3p |
| KAT2A | hsa-miR-3919 | WSB1 | hsa-miR-4646-3p |
| KAT2A | hsa-miR-558 | WSB1 | hsa-miR-5689 |
| KAT2A | hsa-miR-874-5p | WSB1 | hsa-miR-4433b-5p |
| KAT2A | hsa-miR-342-3p | WSB1 | hsa-miR-17-3p |
| TACC3 | hsa-miR-330-3p | WSB1 | hsa-miR-1184 |
| TPX2 | hsa-miR-4326 | WSB1 | hsa-miR-6822-3p |
| TPX2 | hsa-miR-4294 | VHL | hsa-miR-373-5p |
| TPX2 | hsa-miR-1289 | VHL | hsa-miR-616-5p |
| TPX2 | hsa-miR-4282 | VHL | hsa-miR-371b-5p |
| TPX2 | hsa-miR-3065-3p | VHL | hsa-miR-3065-3p |
| TPX2 | hsa-miR-8069 | VHL | hsa-miR-7151-3p |
| TPX2 | hsa-miR-3617-3p | VHL | hsa-miR-3613-3p |
| WSB1 | hsa-miR-5692a | VHL | hsa-miR-513b-5p |
| WSB1 | hsa-miR-3942-5p | VHL | hsa-miR-4755-3p |
| WSB1 | hsa-miR-4703-5p | VHL | hsa-miR-383-3p |
| WSB1 | hsa-miR-10394-5p | VHL | hsa-miR-504-3p |
| WSB1 | hsa-miR-1205 | VHL | hsa-miR-767-5p |
| WSB1 | hsa-miR-5011-5p | VHL | hsa-miR-340-5p |
| WSB1 | hsa-miR-5003-5p | VHL | hsa-miR-320d |
| WSB1 | hsa-miR-3158-5p | VHL | hsa-miR-4429 |
| WSB1 | hsa-miR-3160-5p | VHL | hsa-miR-320c |
| WSB1 | hsa-miR-369-3p | VHL | hsa-miR-320a-3p |
| WSB1 | hsa-miR-4796-5p | VHL | hsa-miR-155-3p |
| WSB1 | hsa-miR-190a-5p | VHL | hsa-miR-320b |
| WSB1 | hsa-miR-9985 | VHL | hsa-miR-4639-3p |
| WSB1 | hsa-miR-3065-5p | VHL | hsa-miR-4482-3p |
| WSB1 | hsa-miR-27a-3p | VHL | hsa-miR-548ar-3p |
| WSB1 | hsa-miR-190b-5p | VHL | hsa-miR-3646 |
| WSB1 | hsa-miR-4282 | VHL | hsa-miR-516a-3p |
| WSB1 | hsa-miR-1277-5p | VHL | hsa-miR-516b-3p |
| WSB1 | hsa-miR-27b-3p | VHL | hsa-miR-7162-5p |

“mRNA”and“miRNA”represent node；“-”represent edge.
