# Supplementary material for: Identification of hypoxia-related diagnostic biomarkers and immune signatures in diminished ovarian reserve
Source: Front Genet. 2025 Aug 4;16:1626992. doi: 10.3389/fgene.2025.1626992 (PMC12358289; doi:10.3389/fgene.2025.1626992)
Supplement: Supplementary file 7 [file Table3.docx]

**Table 3 GSEA analysis results of dataset GSE87201.**

| Description | setSize | enrichmentScore | NES | p.adjust | qvalue |
| --- | --- | --- | --- | --- | --- |
| ZHENG_RESPONSE_TO_ARSENITE_UP | 14 | 0.900235805 | 2.165455936 | 0.01831937 | 0.017932334 |
| REACTOME_RESPONSE_TO_METAL_IONS | 14 | 0.875121133 | 2.105044303 | 0.033872413 | 0.033156785 |
| REACTOME_METALLOTHIONEINS_BIND_METALS | 11 | 0.8990064 | 2.04007762 | 0.033872413 | 0.033156785 |
| KAN_RESPONSE_TO_ARSENIC_TRIOXIDE | 115 | 0.559926721 | 2.034158931 | 0.01831937 | 0.017932334 |
| WP_ZINC_HOMEOSTASIS | 37 | 0.681025371 | 2.029714861 | 0.043981804 | 0.043052593 |
| CHICAS_RB1_TARGETS_LOW_SERUM | 76 | 0.582433651 | 1.982659218 | 0.033872413 | 0.033156785 |
| KEGG_OXIDATIVE_PHOSPHORYLATION | 94 | 0.556990655 | 1.963411386 | 0.01831937 | 0.017932334 |
| REACTOME_RESPIRATORY_ELECTRON_TRANSPORT_ATP_SYNTHESIS_BY_CHEMIOSMOTIC_COUPLING_AND_HEAT_PRODUCTION_BY_UNCOUPLING_PROTEINS | 91 | 0.549144286 | 1.92510825 | 0.033872413 | 0.033156785 |
| WU_CELL_MIGRATION | 166 | 0.497742467 | 1.90202502 | 0.01831937 | 0.017932334 |
| WINTER_HYPOXIA_METAGENE | 218 | 0.42520225 | 1.678488892 | 0.039145939 | 0.038318896 |

GSEA：Gene Set Enrichment Analysis。
