# Supplementary material for: Identification of hypoxia-related diagnostic biomarkers and immune signatures in diminished ovarian reserve
Source: Front Genet. 2025 Aug 4;16:1626992. doi: 10.3389/fgene.2025.1626992 (PMC12358289; doi:10.3389/fgene.2025.1626992)
Supplement: Supplementary file 8 [file Table4.docx]

**Table 4. GSVA enrichment analysis results of GSE87201 between LOR and HOR groups.**

| Description | logFC | AveExpr | t | P.Value |
| --- | --- | --- | --- | --- |
| RAHMAN TP53 TARGETS PHOSPHORYLATED | -0.533802413 | -0.025855568 | -4.645942852 | 0.000147238 |
| REACTOME VITAMIN B2 RIBOFLAVIN METABOLISM | -0.527310225 | -0.194768956 | -3.629708498 | 0.001619226 |
| OHASHI AURKA TARGETS | 0.522554418 | -0.022602876 | 3.606152201 | 0.001711231 |
| REACTOME CAMK IV MEDIATED PHOSPHORYLATION OF CREB | 0.546347723 | -0.008562634 | 3.454970646 | 0.002437124 |
| REACTOME RHOT1 GTPASE CYCLE | 0.557998444 | -0.124905933 | 3.425552049 | 0.00261008 |
| REACTOME SUMO IS CONJUGATED TO E1 UBA2 SAE1 | -0.626955037 | -0.014127024 | -3.235641198 | 0.004053609 |
| REACTOME CREB1 PHOSPHORYLATION THROUGH THE ACTIVATION OF CAMKII CAMKK CAMKIV CASCASDE | 0.563974883 | -0.031996415 | 3.017565357 | 0.006678202 |
| JOHANSSON BRAIN CANCER EARLY VS LATE UP | 0.501075212 | -0.072427213 | 3.006704818 | 0.006844893 |
| GAVIN IL2 RESPONSIVE FOXP3 TARGETS DN | 0.505461477 | 0.007600638 | 2.778561916 | 0.011426774 |
| REACTOME EXTRINSIC PATHWAY OF FIBRIN CLOT FORMATION | -0.559504727 | -0.029689545 | -2.736189251 | 0.012551966 |
| REACTOME SEROTONIN AND MELATONIN BIOSYNTHESIS | -0.505948547 | 0.039226085 | -2.449587793 | 0.023401351 |

GSVA：Gene set variation analysis；LOR：Low ovarian reserve；HOR：High ovarian reserve。
