# Supplementary material for: Identification of hypoxia-related diagnostic biomarkers and immune signatures in diminished ovarian reserve
Source: Front Genet. 2025 Aug 4;16:1626992. doi: 10.3389/fgene.2025.1626992 (PMC12358289; doi:10.3389/fgene.2025.1626992)
Supplement: Supplementary file 9 [file Table6.docx]

**Table S2. mRNA-RBP interaction network nodes.**

| mRNA | RBP | mRNA | RBP |
| --- | --- | --- | --- |
| FANCI | ALYREF | TPX2 | DDX3X |
| FANCI | DDX3X | TPX2 | ELAVL1 |
| FANCI | ELAVL1 | TPX2 | FUS |
| FANCI | FUS | TPX2 | G3BP1 |
| FANCI | G3BP1 | TPX2 | HNRNPC |
| FANCI | HNRNPC | TPX2 | IGF2BP1 |
| FANCI | PTBP1 | TPX2 | IGF2BP3 |
| FANCI | RBFOX2 | TPX2 | LIN28B |
| FANCI | RBMX | TPX2 | RBFOX2 |
| FANCI | RNPS1 | TPX2 | RBMX |
| FANCI | TARDBP | TPX2 | RNPS1 |
| FANCI | U2AF1 | TPX2 | TARDBP |
| FANCI | U2AF2 | TPX2 | U2AF2 |
| FANCI | UPF1 | TPX2 | YTHDC1 |
| KAT2A | ALYREF | TPX2 | YTHDF1 |
| KAT2A | DDX3X | TPX2 | YTHDF2 |
| KAT2A | RBMX | VHL | ALYREF |
| KAT2A | RNPS1 | VHL | DDX3X |
| KAT2A | U2AF1 | VHL | ELAVL1 |
| KAT2A | U2AF2 | VHL | HNRNPC |
| KAT2A | YTHDC1 | VHL | IGF2BP2 |
| KAT2A | YTHDF1 | VHL | LIN28B |
| TACC3 | ALYREF | VHL | RBMX |
| TACC3 | DDX3X | VHL | TARDBP |
| TACC3 | ELAVL1 | VHL | U2AF1 |
| TACC3 | FUS | VHL | U2AF2 |
| TACC3 | G3BP1 | VHL | UPF1 |
| TACC3 | HNRNPC | VHL | YTHDF1 |
| TACC3 | HNRNPK | WSB1 | ALYREF |
| TACC3 | RBFOX2 | WSB1 | DDX3X |
| TACC3 | RBMX | WSB1 | ELAVL1 |
| TACC3 | RNPS1 | WSB1 | FUS |
| TACC3 | SRSF1 | WSB1 | HNRNPC |
| TACC3 | TARDBP | WSB1 | RBMX |
| TACC3 | U2AF1 | WSB1 | RNPS1 |
| TACC3 | U2AF2 | WSB1 | TARDBP |
| TACC3 | YTHDC1 | WSB1 | U2AF1 |
| TACC3 | YTHDF1 | WSB1 | U2AF2 |
| TPX2 | ALYREF |  |  |

“mRNA”and“RBP”represent node，“-”represent edge。RBP：RNA binding protein。
