# Supplementary material for: Identification of hypoxia-related diagnostic biomarkers and immune signatures in diminished ovarian reserve
Source: Front Genet. 2025 Aug 4;16:1626992. doi: 10.3389/fgene.2025.1626992 (PMC12358289; doi:10.3389/fgene.2025.1626992)
Supplement: Supplementary file 10 [file Table10.docx]

**Table 6. KEGG enrichment analysis results of hub genes.**

| Ontology | ID | Description | GeneRatio | BgRatio | pvalue | p.adjust |
| --- | --- | --- | --- | --- | --- | --- |
| KEGG | hsa03460 | Fanconi anemia pathway | 1/3 | 54/8164 | 0.0197 | 0.0566 |
| KEGG | hsa04330 | Notch signaling pathway | 1/3 | 59/8164 | 0.0215 | 0.0566 |
| KEGG | hsa03250 | Viral life cycle - HIV-1 | 1/3 | 63/8164 | 0.0230 | 0.0566 |
| KEGG | hsa05211 | Renal cell carcinoma | 1/3 | 69/8164 | 0.0251 | 0.0566 |
| KEGG | hsa04066 | HIF-1 signaling pathway | 1/3 | 109/8164 | 0.0395 | 0.0657 |

KEGG：Kyoto encyclopedia of genes and genomes。
